# Asymmetric Effects of Weather-Integrated Human Brucellosis Forecasting System Using a New Nonlinear Autoregressive Distributed Lag Model

**DOI:** 10.1155/2024/8381548

**Published:** 2024-03-05

**Authors:** Yongbin Wang, Chenlu Xue, Bingjie Zhang, Yuchun Li, Chunjie Xu

**Affiliations:** ^1^Department of Epidemiology and Health Statistics, School of Public Health, Xinxiang Medical University, No. 601 Jinsui Road, Hongqi District, Xinxiang 453003, Henan Province, China; ^2^Beijing Key Laboratory of Antimicrobial Agents/Laboratory of Pharmacology, Institute of Medicinal Biotechnology, Chinese Academy of Medical Sciences and Peking Union Medical College, Beijing 100050, China

## Abstract

Human brucellosis (HB) remains a significant public health concern in China. This study aimed to investigate the long- and short-term asymmetric impacts of meteorological variables on HB and develop an early prediction system. Monthly data on HB incidence and meteorological variables were collected from 2005 to 2020. The study employed the autoregressive distributed lag (ARDL) and nonlinear ARDL (NARDL) to analyze the long- and short-term effects of climate variables on HB. Subsequently, the data were split into training (from January 2005 to December 2019) and testing parts (from January to December 2020) to develop and validate the forecasting accuracy of both models. During 2005–2020, there were 34,993 HB cases (2.03 per 100,000 persons) and there was an overall rising trend (average annual percentage change = 21.18%, 95%CI 18.36%–26.01%) in HB incidence, peaked in May and troughed in December per year. A 1 m/s increment and decrement in differenced (*Δ*) average wind velocity (AWV) contributed to 73.8% and 87.5% increases in *Δ*HB incidence, respectively (Wald long-run asymmetry test (WLR) = 1.17, *P*=0.25). A 1 hr increment and decrement in *Δ*(average relative humidity) contributed to both 3.1% increases in *Δ*HB incidence (Wald short-run asymmetry test = 3.01, *P*=0.003). Average temperature (AT) (*P* < 0.001) and average air pressure (*P*=0.012) played a long-run linear impact on HB. *Δ*(aggregate precipitation) (WLR = 1.76, *P*=0.08) and *Δ*(aggregate sunshine hours) (WLR = 0.07, *P*=0.94) did not have a significant long-term asymmetric impact on *Δ*log(HB). *ΔΔ*AT(+) and *ΔΔ*AWV(−) at a 1-month lag had a meaningful short-run effect on *Δ*log(HB). In the forecasting aspect, the NARDL produced significantly smaller error rates compared to the ARDL. Weather variability played significant long- and short-run asymmetric roles in HB incidence. The NARDL by integrating climatic variables could accurately capture the dynamic structure of HB epidemic, meaning that meteorological variables should be integrated into the public health intervention plan for HB.

## 1. Background

Human brucellosis (HB) is a bacterial zoonosis caused by *Brucella* spp., primarily infecting cattle, swine, goats, sheep, and dogs. It mainly occurs by contact with infected animals or their products [1, 2]. Although significant progress has been made in controlling HB in many countries, the global burden remains substantial, with over 500,000 new cases reported annually. The disease has serious health implications and socioeconomic impacts [3, 4]. In China, the number of HB cases declined from 47,139 in 2016 to 37,947 in 2018, but there was a subsequent rebound in 2019 [5]. According to the latest data released by the Chinese CDC, the number of HB reached 75,858 cases in 2023 [6]. In response to the National Brucellosis Prevention and Control Plan aiming to manage HB in both animals and humans across China [7], it is critical to accurately identify factors influencing HB and construct effective forecasting models for health interventions.

Numerous factors contribute to HB incidence, encompassing the geographical environment, consumption of unpasteurized milk products, and engagement in high-risk occupations [4, 8, 9]. Also, in the context of global climate change, the impact of climatic variables on the transmission of infectious diseases has garnered significant attention [10, 11]. Meteorological factors are believed to influence pathogenic agent growth, host population dynamics, and human behaviors [10]. Consequently, these variables may serve as early indicators of infectious disease risk. Previous research has established correlations between climatic variables and the risk of HB transmission [4, 8, 9]. For example, Sun et al. [12] indicated that temperature at the lags of 0, 2, and 3 months and relative humidity at a 0-month lag were significantly related to HB transmission in China using a spatial panel model. Cao et al. [8] found that air pressure at a 2-month lag, wind speed at a 1-month lag, and temperature at a 2-month lag were significantly associated with HB incidence using a linear autoregressive integrated moving average (ARIMA) model. Yang et al. [13] suggested that the seasonality of HB was significantly associated with air pressure, rainfall, and temperature using a distribution lag nonliner model (DLNM). However, there has been limited evidence about sunshine and wind on HB, and prior studies typically operated within a linear framework and overlooked the exploration of long- and short-term asymmetric dynamic impacts of meteorological variables on HB [4, 8, 9]. This refers to situations where increases or decreases in meteorological factors lead to distinct effects, holding greater practical significance for HB prevention and control. Additionally, past research failed to consider robust autocorrelations among dependent variables, leading to potential overestimations in time series analysis. The DLNM and artificial neural networks (ANNs) are currently the most commonly used nonlinear models for analyzing the associations between meteorological factors and diseases or forecasting the epidemics of diseases, but these models fail to explore both long- and short-term effects of the variables on outcomes simultaneously. Therefore, this study aimed to address these gaps by introducing a new nonlinear autoregressive distributed lag (NARDL) model, known for its enumerated advantages [14–16]: (1) facilitating examination of long- and short-term asymmetries, (2) enabling time series with varying orders of integration, (3) prioritizing resolution of endogenous issues between variables, and (4) automatically incorporating autocorrelations in time series analysis.

During the past decade, endemic regions of HB gradually spread from north of China to some southern provinces, including Henan [2, 17]. Here, we carried out a population-based time series study aiming to investigate the long- and short-run asymmetric dynamic associations between meteorological factors and HB in Henan by use of NARDL and to determine whether the NARDL can improve the forecasting accuracy of HB epidemic over the autoregressive distributed lag (ARDL). Such an analysis that delves into the intrinsic relationship between climatic factors and HB (i.e., understanding the varying effects of increases or decreases in climatic factors, as well as how potential factors respond to short-term changes and evolve over time) is critical for providing comprehensive insights into controlling HB epidemic in Henan.

## 2. Materials and Methods

### 2.1. HB Data

Henan is a province in Central China covering an area of 167,000 km^2^. It is the largest province with a registered population in China, with a population of 115 million in 2022. Henan is mostly located in the warm temperate zone, the south trans-subtropical, and belongs to a continental monsoon climate from the north subtropical to the warm temperate zone (Figure S1).

The monthly HB incidence in Henan from 2005 to 2020 was extracted from the Data-Center of China Public Health Science (DCPHS) operated by the Chinese CDC and the Health Commission of Henan Province. The population data during the same period was from the Henan Statistical Yearbook. All HB incidents were confirmed by authorized institutions and professionals according to the diagnostic criteria for HB (http://www.nhc.gov.cn/wjw/s9491/wsbz.shtml).

### 2.2. Meteorological Data

The daily meteorological variables, including average temperature (AT), average air pressure (AAP), aggregate precipitation (AP), aggregate sunshine hours (ASH), average relative humidity (ARH), and average wind velocity (AWV), were provided by the National Meteorological Science Data Center (http://data.cma.cn/). To address missing data, we additionally utilized information from the Huiju Data website (http://hz.hjhj-e.com/home/meteorologicalData/dataDetailsThreeYear/) for supplementation. Subsequently, these variables were compiled into a monthly time series format.

### 2.3. Statistical Analysis

During statistical description, all study variables were represented as mean ± standard deviation ($\overset{―}{x}\pm s$). Average annual percentage change (AAPC) was computed to describe the epidemiological change trend of HB [18]. Spearman's correlation was applied to test the correlation between meteorological factors and HB, and a correlation coefficient greater than 0.9 or variance inflation factor (VIF) greater than 10 was indicative of a strong collinearity between variables [19, 20]. If there was multicollinearity between variables, and then these variables were entered into different NARDL and ARDL models with other meteorological drivers to investigate their effects on HB.

ARDL has been used to deal with problems of autocorrelations and nonstationarity of key variables, and our prior study has detailed this model [21]. However, the ARDL frequently yields a biased result due to the presence of nonlinear and/or asymmetric impacts in consideration of meteorological factors on diseases [22]. The NARDL was thus introduced to overcome the weakness. In NARDL, the term “autoregressive” refers to the inclusion of lagged values of HB incidence itself. The “nonlinear” aspect indicates that the relationship between HB and weather variables can be nonlinear, and “distributed lag” signifies that current values of HB incidence are influenced by both its past values and the past values of weather factors. This method also allows investigating the long- and short-term asymmetric dynamic effects [15, 23]. In the presence of asymmetric impacts, the NARDL can quantify the responses of HB incidence to positive and negative changes in each of the meteorological factors by taking into account the positive and negative partial sums of increments and decrements in these variables [15, 23]. The NARDL involves four steps [14, 15, 23]: first, investigation of the order of integration. Although the NARDL has relaxed the integration requirement, the order of integration cannot be greater than one [14]. Besides, a pseudo regression may be produced by the nonstationary regressors. Thus, the augmented Dickey–Fuller statistic was chosen to test the order of integration and stationarity in independent and dependent variables [24]. If the results indicated a nonstationary series, logarithmic transformation and/or differencing (*Δ*) were applied to achieve stationarity. Second, investigation of the long-run asymmetric cointegration. To check whether there was a long-run asymmetric cointegration between regressors and dependent variables, the bounds test (F statistic) was applied [25]. If evidence pointed to the presence of such a relationship, then a Wald test was used to investigate the short- and long-term asymmetries [14, 15]. Third, effect estimation. The positive and negative dynamic multiplier effect of regressors on the dependent variable could be estimated [26]. Finally, forecasting. The data between January 2005 and December 2019 were treated as the training set, and the remaining as the testing set. To demonstrate the forecasting capacity of the NARDL for the HB epidemics by integrating meteorological factors, the error rate metrics, including mean absolute deviation (MAD), root mean square error (RMSE), mean error rate (MER), mean absolute percentage error (MAPE), and root mean square percentage error (RMSPE) were computed to assess the forecasting accuracy of the NARDL and ARDL by use of the modified Diebold–Mariano (DM) test [27]:

(1) $$\begin{array}{c} \text{MAD}=\frac{1}{N}\sum_{i=1}^{N}\left|X_{i}-{\hat{X}}_{i}\right|, \end{array}$$

(2) $$\begin{array}{c} \text{RMSE}=\sqrt{\frac{1}{N}\sum_{i=1}^{N}{\left(X_{i}-{\hat{X}}_{i}\right)}^{2}}, \end{array}$$

(3) $$\begin{array}{c} \text{MER}=\frac{\frac{1}{N}\sum_{i=1}^{N}\left|X_{i}-{\hat{X}}_{i}\right|}{{\overset{―}{X}}_{i}}, \end{array}$$

(4) $$\begin{array}{c} \text{MAPE}=\frac{1}{N}\sum_{i=1}^{N}\frac{\left|X_{i}-{\hat{X}}_{i}\right|}{X_{i}}\times 100, \end{array}$$

(5) $$\begin{array}{c} \text{RMSPE}=\frac{1}{N}\sqrt{\sum_{i=1}^{N}{\left(\frac{X_{i}-{\hat{X}}_{i}}{X_{i}}\right)}^{2}}. \end{array}$$

In this study, the notation of the NARDL is calculated as follows:

(6) $$\begin{array}{c} \Delta \text{ Log}\left(Y_{t}\right)=a_{0}+\sum_{i=1}^{p_{i}}p_{1i}\Delta Y_{t-1}+\sum_{i=0}^{q_{1}}q_{1i}^{+}x_{t-1}^{+}+\sum_{i=0}^{q_{2}}q_{2i}^{-}x_{t-1}^{-}+\sum_{i=1}^{p_{2}}p_{2i}\Delta Y_{t-i}+\sum_{i=0}^{q_{3}}q_{3i}^{+}\Delta x_{t-i}^{+}\sum_{i=0}^{q_{4}}q_{4i}^{-}\Delta x_{t-i}^{-}+a_{1}\text{month}+\epsilon_{t}, \end{array}$$

where *Y*_*t*_ represents HB cases, *x* signifies the meteorological factors (e.g., AT, AAP, AP, ASH, ARH, and AWV), *x*^+^ and *x*^−^ are the positive and negative partial sums of increases and decreases in each meteorological factor, respectively (which quantifies the long-term asymmetric impact), *p* and *q* denote the optimal lag orders of HB cases and meteorological variables, respectively (which quantities the short-term asymmetric impact), month represents the seasonal variables, and *Δ* refers to the first-order difference.

In this study, the maximum lag orders were specified as 3 because there is an about 2−4-week incubation period from HB infection to onset of symptoms [1] and maximum 2-month delay from symptom appearance to clinical diagnosis in China [28], and then the optimal lag orders were determined by Akaike information criterion (AIC), Schwarz criterion (BIC), Hannan–Quinn (HQ) criterion, log-likelihood, and adjusted *R*^2^. The autocorrelation in the dependent variable was determined by the partial autocorrelation function (PACF) plot [29], which indicates the correlation between the current observations and the past observations under the condition of given cases. The 11 monthly dummy variables were included in the model to adjust for the seasonal effect. The long-term trend was also handled in the equation by differencing all of the variables. Additionally, the stability of the NARDL was tested using the cumulative sum (CUSUM) statistics [25]. All statistical analyses were performed by EViews 10 (IHS, Inc., USA) and R 4.2.0 (R Development 164 Core Team, Vienna, Austria), and a two-sided *P* ≤ 0.05 was considered significant.

## 3. Results

### 3.1. Statistical Description

In the period 2005–2020, a total of 34,993 HB cases (2.03 per 100,000 persons) were reported in Henan, on average with the number of monthly and annualized 194 and 2,333 cases, respectively. Overall, the epidemic trend in HB incidence rose during the study period (AAPC = 21.18%, 95%CI 18.36%–26.01%), peaking in 2015, with 5,897 case notifications (5.26 per 100,000 persons), and then a downward trend was going until 2018 when the number of reported cases was 2,144 (1.87 per 100,000 persons), and there was a slight rebound in early 2020 with 3,202 case notifications (2.78 per 100,000 persons). Besides, the HB incidence represented obvious periodic and seasonal characteristics. There was a peak in May and a trough in the winter of each year.

**Figure 1:**
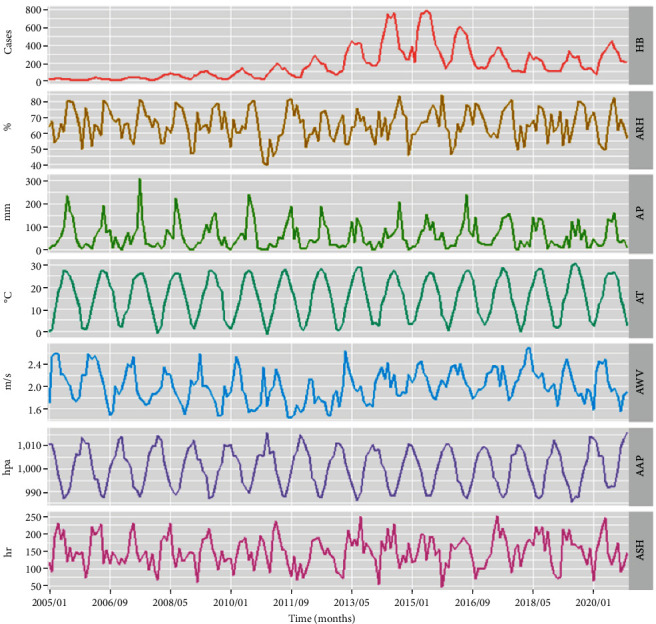
Changing trend plot of the climatic variables and HB cases in Henan, 2005–2020. HB, human brucellosis; ARH, average relative humidity; AP, aggregate precipitation; AT, average temperature; AWV, average wind velocity; AAP, average air pressure; and ASH, aggregate sunshine hours.

**Figure 2:**
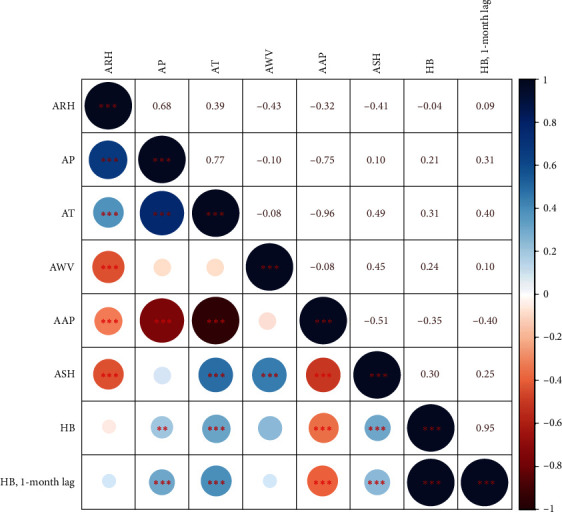
Spearman's correlation among variables. It was seen that there is a strong collinearity between AAP and AT. HB, human brucellosis; ARH, average relative humidity; AP, aggregate precipitation; AT, average temperature; AWV, average wind velocity; AAP, average air pressure; and ASH, aggregate sunshine hours.

Summary statistics for monthly HB cases and meteorological factors were described in Table 1. The means of ARH, AP, AT, AWV, AAP, and ASH were 65.61 ± 9.68%, 60.52 ± 58.15 mm, 15.49 ± 9.36°C, 2.01 ± 0.30 m/s, 1,000.36 ± 8.47 hPa, and 149.46 ± 44.20 hr, respectively. As shown in Figure 1, seemingly the same changing trend was observed between HB and AP, AT, AWV, and ASH. However, a contrary trend was found between ARH and AAP. Additionally, strong collinearity was revealed between AAP and AT due to Spearman's correlation coefficient greater than 0.9 and VIF greater than 10 (Table 1 and Figure 2).

### 3.2. The Asymmetric and Symmetric Effects of Meteorological Factors on HB

**Figure 3:**
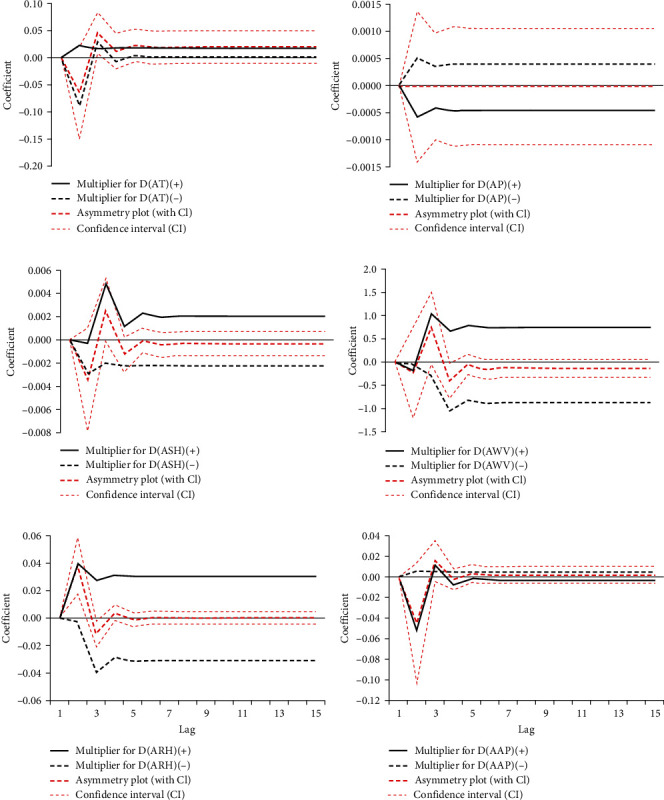
Dynamic multiplier asymmetric effect of climatic variables on HB: (a) multiplier graph for AT; (b) multiplier graph for AP; (c) multiplier graph for ASH; (d) multiplier graph for AWV; (e) multiplier graph for ARH; and (f) multiplier graph for AAP. AT, average temperature; AP, aggregate precipitation; ASH, aggregate sunshine hours; AWV, average wind velocity; ARH, average relative humidity; AAP, average air pressure; and CI, confidence interval.

Based on the modeling process of ARDL and NARDL models, the NARDL (1, 0, 1, 0, 0, 2, 0, 1, 2, 0, 1) and ARDL (1, 0, 0, 0, 2, 1) were determined as the best possible models (see the details of the development of NARDL and ARDL models in Figures S2–S5 and Tables S1 and S2). As shown in Table 2, there was statistical significance in the long-run coefficients of AWV and ARH, which were positively related to HB. When *Δ*AWV increased by 1 m/s, *Δ*HB increased approximately by 73.8%, and when it reduced by 1 m/s, *Δ*HB increased approximately by 87.5%, with the increase in *Δ*HB being the cumulative increase. When *Δ*ARH increased and reduced by 1%, respectively, *Δ*HB increased approximately by about 3.1%, with the increase in *Δ*HB being the cumulative increase. *Δ*AT, *Δ*AP, *Δ*ASH, and *Δ*AAP were associated with a nonsignificant long-term coefficient. However, as shown by results from the ARDL model, *Δ*AT and *Δ*AAP were shown to have a positive significant long-term coefficient (Table 2), corroborating the long-term linear effect on *Δ*HB, when *Δ*AT and *Δ*AAP increased by 1°C and 1 hPa, *Δ*HB increased approximately by 8.1% and decreased by 8.4%, respectively. *Δ*AT(−) had a meaningful short-run positive effect on *Δ*HB; *Δ*AWV(−) at a 1-month lag had a meaningful short-run negative effect on *Δ*log(HB).  Table 3 details the Wald test results for asymmetry, suggesting that *Δ*AAP might have a long-run asymmetric impact on *Δ*HB, which was also confirmed by the dynamic multiplier plot (Figure 3(a)−3(f)), yet the long-term coefficient was nonsignificant. A long-run asymmetric relationship was not observed for *Δ*AT, *Δ*AP, *Δ*ASH, *Δ*ARH, and *Δ*AWV. Besides, *Δ*AT and *Δ*ARH might have a short-run asymmetric impact on *Δ*HB.

### 3.3. Forecasting HB Epidemic

**Figure 4:**
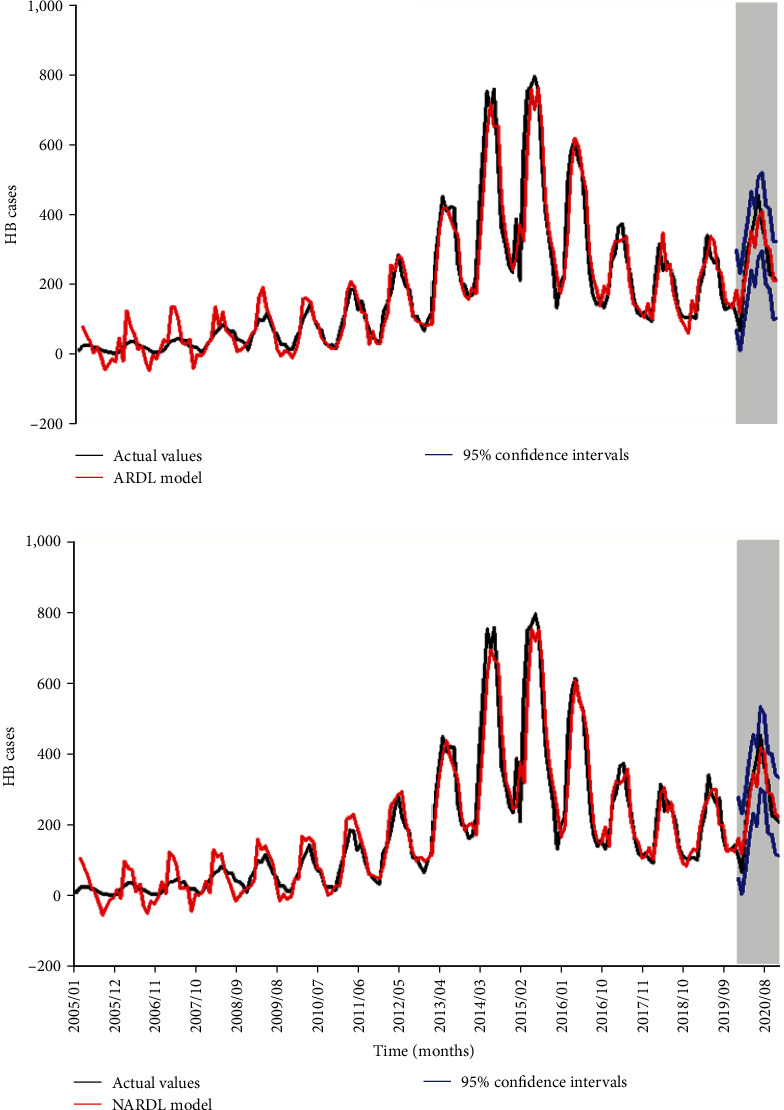
Comparison of the actual observations and predictive values based on both models: (a) results from the ARDL model and (b) results from the NARDL model. ARDL, autoregressive distributed lag model; NARDL, nonlinear autoregressive distributed lag model.

By developing the best possible ARDL and NARDL on the training set, and then forecasting the remaining data. The fitting and predicative results are depicted in Figure 4, and the performance comparison is summarized in Table 4. It was found that the NARDL produced lower error rates than those of the ARDL in both fitting and predictive aspects, and the DM test was significant in the predictive part, meaning that the predictive capacity of NARDL significantly outperformed the ARDL. This demonstrated that the NARDL was better able to capture dynamic dependency characteristics in HB incidence.

## 4. Discussion

This study discovered that, from a long-run perspective, AWV and ARH might have a significant positive nonlinear association with HB after adjustment for seasonality, autocorrelation, and time variable. AT and AAP might have a linear positive association with HB. From a short-run perspective, AT(−) might be positively associated with HB, and AWV(−) at a 1-month lag might be reversely associated with HB. To the best of our knowledge, this is the only study to investigate the long- and short-run asymmetric impacts of meteorological factors on HB and establish an early forecasting system using the ARDL and NARDL. Our results corroborated the lead time, the asymmetric and symmetric impacts of meteorological parameters on HB, along with the usefulness of the NARDL in capturing the dynamic epidemic structure in HB incidence. These findings are helpful in estimating the epidemic trajectory of HB, giving enough time to develop targeted prevention and control policies and to implement public health interventions.

Weather-integrated infectious disease prediction models predominantly include ARDL [21], generalized linear models [30], Bayesian structural time series [31], and ARIMA [32]. In comparison to the aforementioned models, the NARDL offers several advantages in modeling HB incidence series [14–16, 33]: (1) NARDL can account for cases where the impact of positive changes in weather factors differs from the impact of negative changes; (2) by including lagged values of variables in the model, NARDL enables the examination of both immediate and persistent effects of weather factors, contributing to a more comprehensive analysis; and (3) NARDL allows for straightforward interpretation of coefficients, making it possible to capture the direction and magnitude of the effects of weather factors. This enhances understanding and facilitates informed policy and decision-making; (4) the incorporation of nonlinear and asymmetric terms in NARDL improves model fit by better capturing the underlying dynamics of the data, leading to more accurate and reliable predictions. These qualities equip NARDL to better elucidate the relationships between HB and weather factors in real-world scenarios. Moreover, the NARDL has shown successful applications in studying the relationships between macroeconomic variables, financial indicators, and other economic factors in economics and finance research. Therefore, it appears that the weather-integrated NARDL model holds promise for analyzing and forecasting HB epidemics in other regions and similar phenomena (e.g., other infectious diseases). Also, future research should concentrate on the comparison of the forecasting ability between NARDL and ANNs (e.g., long- and short-term memory neural network, neural network nonlinear autoregression, and generalized regression neural network).

Our results revealed that overall a rising trend was observed in HB incidence, aligned with the overall epidemic trend worldwide and in China [3, 34]. This might be explained by the rising demand for meat consumption, the expansion of animal industries, urbanization, the lack of hygienic measures and vaccinations in animal husbandry, as well as the failure to remove infected animals [3]. Besides, we found an obvious seasonal profile in HB morbidity, with a peak in May and a trough in December, in alignment with the seasonality at the national level of China [3]. The strong seasonal profile may be closely associated with the peak period for abortions and parturitions among livestock in the spring and summer [3].

An interesting finding is that AWV might be one of the most important contributors to HB, which was shown to have a significant long-term positive effect on HB, seemingly a reduction in AWV has a stronger effect than an increase, and this relationship tended to be symmetric, whereas a significant short-run negative effect was found in AWV at a 1-month lag. Previous work documented that AWV was positively related to respiratory infectious disease (e.g., scarlet fever and mumps) [8, 21], these studies provide additional support for our finding. The fact that there is more wind in spring and summer compared to other seasons in Henan also seems to adequately account for the high-risk seasonality in HB incidence in May each year. Plausible explanations for our long- and short-run findings are as follows [8, 35, 36]: (1) *Brucella* live shorter in the air with high-speed wind in the short run; (2) the higher the wind speed, the greater evaporation, which in turn indirectly affected brucellosis by evaporation and ultimately had a strong driving effect on HB in the long run; (3) sheep and goats are the main sources of infection, and as animal husbandry has developed in windy and dry climate in northern China, there is a higher danger of contracting brucellosis in the long run; and (4) higher wind speeds facilitate the greater spread of pollutants carrying *Brucella*, increasing transmission between livestock populations, further increasing the risk to humans in the long run.

Another important finding is that ARH might have a significant long-term positive effect on HB, and seemingly the increase has the same effect as an increase in HB. From a short-run perspective, this relationship tended to be symmetric. Our results share a similarity with several studies that indicated a positive relationship between ARH and other respiratory contagious disease (e.g., scarlet fever and mumps) [21, 29]. However, in contradiction with our conclusions, several studies also reported a reverse relationship between ARH and HB [34, 37]. It may be speculated that this discrepancy is explained in part by the different models used to analyze the data or different regions or by no adjustment for autoregression in the dependent variable. Nevertheless, our finding is consistent with a previous study indicating that high humidity are as conducive to the long-term survival of *Brucella* as many other pathogens in the environment, which could be explained with that high humidity may increase the risk of exposure to this pathogen so as to increase the HB incidence [38].

The third important finding is that AAP played an asymmetric impact on HB, but the long-run coefficient was not significant. The significant coefficient in ARDL indicated a weak increase approximately by 8.4% in *Δ*HB when *Δ*AAP decreased or increased by 1 hPa. It is consistent with the finding that atmosphere pressure was negatively correlated with HB [32]. A study suggested that atmospheric pressure may indirectly affect HB through temperature and rainfall [8]. Warm temperature would force the air near the ground to move upward, causing low pressure in the local area which is usually accompanied by rainfall events, leading to increased indoor contact between humans and livestock. However, our research does not match with a study observing that an increase in air pressure aggravates the disease [35]. Perhaps because different regions have led to different results. Besides, very few studies have clarified the relationship between atmosphere pressure and HB, thus further investigation is needed to clarify the mechanism.

The fourth important finding is that AT played a long-run linear impact on HB, indicating a weak increase approximately by 8.1% in *Δ*HB when *Δ*AT increased or decreased by 1°C. In the short-run, a significant positive effect was found in AT and this relationship tended to be symmetric. On one hand, temperature as an environmental factor affects the condition for the survival of *Brucella*, which increases the risk of bacterial transmission [36, 39]. On the other hand, higher temperature in late spring and early summer increase husbandry activities for sheep and goats, including shearing, breeding, processing of meat products, and commercialization of sheep products, consequently increasing the opportunities for humans to contact susceptible animals or contaminated animal products [36, 39, 40]. In addition, researchers also found ASH [32, 34, 37] and AP [37, 39] correlated with the HB incidence, whereas we did not indicate a significant long- and short-term relationship. Therefore, further validation work is expected to go on in other areas.

Our research focused on the long- and short-run asymmetric and/or symmetric impacts of variations in meteorological factors on HB and integration of this effect into the HB early prediction system. Prior study has emphasized the significance of considering the changes in population immunity, autocorrelations, a series of possible lags and relationship patterns, seasonality, and long-term trend when performing a time series analysis [41]. Except for the changes in population immunity that we failed to fully investigate due to a lack of data, other problems were taken into account. Therefore, we are confident that we provide valid and trustworthy evidence: variation in meteorological factors plays a crucial long- and short-run asymmetric and/or symmetric role in HB incidence and the NARDL is a useful aid for forecasting HB epidemic. Also, our work has some limitations. First, underreporting or underdiagnosis is inevitable for a passive monitoring system. Second, this study is an ecological trend study that does not allow for an investigation into the individual-based relationship and infer a causal effect. Third, the findings relied on data from Henan, it is necessary to verify whether the model can be generalized to predict HB epidemic in other regions or other infectious diseases. Fifth, the outbreak of COVID-19 has already impacted the epidemiological trends of many infectious diseases. Whether it affects the predictive accuracy of the NARDL model warrants further investigation. Finally, we do not control for the effect of the unmeasured confounders (e.g., geographic and socioeconomic factors, population density, and host susceptibility).

## 5. Conclusion

We discovered that AWV, ARH, AT, and AAP play an important long- and short-term asymmetric and/or symmetric role in HB incidence. In the context of global climate change, meteorological variables should be included in the public health intervention plan for HB. The long- and short-term asymmetric effects of weather-integrated NARDL are better suited to capture the dynamic epidemic structure in HB compared to the ARDL, which can be regarded as a useful tool for guiding HB prevention and control.

## Figures and Tables

**Table 1:** Statistical descriptions for monthly HB cases and climatic factors in Henan, 2005–2020.

Variable	Mean	SD	Min	*P*_25_	*P*_50_	*P*_75_	Max	VIF
HB cases	182.26	182.62	0.00	38.00	119.50	254.25	794.00	—
ARH	65.61	9.68	39.40	59.43	65.38	73.53	84.50	3.47
AP	60.52	58.15	0.31	17.33	41.17	87.97	307.40	3.17
AT	15.49	9.36	−1.23	6.91	16.48	24.69	30.90	18.62
AWV	2.01	0.30	1.43	1.80	1.99	2.21	2.70	2.15
AAP	1,000.36	8.47	985.33	992.35	1,001.36	1,007.55	1,016.20	14.82
ASH	149.46	44.20	46.83	118.53	148.13	179.59	250.90	4.30
HB cases, 1-month lag	—	—	—	—	—	—	—	1.26

ARH, average relative humidity; AP, aggregate precipitation; AT, average temperature; AWV, average wind velocity; AAP, average air pressure; ASH, aggregate sunshine hours; HB, human brucellosis; and VIF, variance inflation factor.

**Table 2:** Long- and short-term estimates of the best NARDL and ARDL methods.

NARDL model			ARDL model		
Variable	Coefficient	*P*	Variable	Coefficient	*P*
Long-run estimate			Long-run estimate		
*Δ*AT(+)	0.018	0.603	*Δ*AT	0.081	<0.001
*Δ*AT(−)	−0.001	0.973	*Δ*AP	−0.0004	0.677
*Δ*AP(+)	−0.0004	0.696	*Δ*ASH	0.001	0.530
*Δ*AP(−)	−0.0004	0.693	*Δ*AWV	1.449	0.022
*Δ*ASH(+)	0.002	0.376	*Δ*ARH	0.030	0.004
*Δ*ASH(−)	0.002	0.290	*Δ*AAP	−0.084	0.012
*Δ*AWV(+)	0.738	0.044	Short-run estimate		
*Δ*AWV(−)	0.875	0.031	*ΔΔ*AWV	0.137	0.691
*Δ*ARH(+)	0.031	0.001	*ΔΔ*AWV, 1-month lag	−0.648	0.050
*Δ*ARH(−)	0.031	0.002	*ΔΔ*ARH	0.027	0.007
*Δ*AAP(+)	−0.003	0.913			
*Δ*AAP(−)	−0.005	0.846			
Short-run estimate					
*ΔΔ*AT(−)	0.089	0.029			
*ΔΔ*ASH(+)	0.000	0.889			
*ΔΔ*ASH(+), 1-month lag	0.002	0.168			
*ΔΔ*AWV(+)	−0.177	0.671			
*ΔΔ*AWV(−)	0.047	0.909			
*ΔΔ*AWV(−), 1-month lag	−0.809	0.045			
*ΔΔ*ARH(−)	0.002	0.850			

Adjustment for seasonality as a dummy variable. NARDL, nonlinear autoregressive distributed lag model; ARDL, autoregressive distributed lag model; AT, average temperature; AP, aggregate precipitation; ASH, aggregate sunshine hours; AWV, average wind velocity; ARH, average relative humidity; AAP, average air pressure; HB, human brucellosis; and VIF, variance inflation factor.

**Table 3:** Long- and short-term asymmetry results using Wald test.

Variable	Long-term asymmetry		Short-term asymmetry	
	WLR	*P*	WSR	*P*
*Δ*AT	0.25	0.78	2.20	0.03
*Δ*AP	0.04	0.97	—	—
*Δ*ASH	0.54	0.59	−0.57	0.57
*Δ*AWV	1.17	0.25	0.95	0.34
*Δ*ARH	−0.04	0.97	3.01	0.003
*Δ*AAP	−2.14	0.03	−0.45	0.66

WLR, Wald long-run asymmetry test; WSR, Wald short-run asymmetry test; AT, average temperature; AP, aggregate precipitation; ASH, aggregate sunshine hours; AWV, average wind velocity; ARH, average relative humidity; AAP, average air pressure.

**Table 4:** Comparison of predictive performance between ARDL and NARDL.

Models	MAD	MAPE	RMSE	MER	RMSPE
ARDL	37.98	20.66	48.01	0.14	0.32
NARDL	33.34	18.49	40.36	0.13	0.28
DM	8.58	5.64	9.70	—	5.12
*P*	<0.001	<0.001	<0.001	—	<0.001

NARDL, nonlinear autoregressive distributed lag model; ARDL, autoregressive distributed lag model; MAD, mean absolute deviation; MAPE, mean absolute percentage error; RMSE, root mean square error; MER, mean error rate; RMSPE, root mean square percentage error; DM, Diebold–Mariano.

## Data Availability

All data for this work are presented in the results and conclusions or please contact the corresponding author on the reproducibility of this work.

## Supplementary Materials

Supplementary Materials Figure S1: geographic snapshot of Henan province. Figure S2: partial autocorrelogram for the differenced HB cases. The presence of local maximum values at 1- and 12-month delays, suggesting the first-order autocorrelation between HB cases. PACF partial autocorrelation function. Figure S3: akaike information criterion (AIC) of top 20 NARDL models. Figure S4: CUSUM graph for the stability test of the NARDL model. The residuals at different time points fell into the 95% confidence intervals, substantiating that the model is stable and valid. CUSUM cumulative sum. Figure S5: akaike information criterion (AIC) of top 20 ARDL models. Table S1: possible NARDL candidates. Table S2: possible ARDL candidates.

## References

[1] World Health Organization (2024). Brucellosis. https://www.who.int/news-room/fact-sheets/detail/brucellosis.

[2] Lai S., Zhou H., Xiong W. (2017). Changing epidemiology of human brucellosis, China, 1955–2014. *Emerging Infectious Diseases*.

[3] Lai S., Chen Q., Li Z. (2021). Human brucellosis: an ongoing global health challenge. *China CDC Weekly*.

[4] Pakzad R., Pakzad I., Safiri S. (2018). Spatiotemporal analysis of brucellosis incidence in Iran from 2011 to 2014 using GIS. *International Journal of Infectious Diseases*.

[5] Tao Z., Chen Q., Chen Y. (2021). Epidemiological characteristics of human brucellosis—China, 2016-2019. *China CDC Weekly*.

[6] Chinese Center for Disease Control and Prevention (2024). Notifiable infectious diseases. https://www.chinacdc.cn/.

[7] Ministry of Agriculture and Rural Affairs of China (2024). National brucellosis control program, 2016-2020. http://www.moa.gov.cn/.

[8] Cao L.-T., Liu H.-H., Li J., Yin X.-D., Duan Y., Wang J. (2020). Relationship of meteorological factors and human brucellosis in Hebei province, China. *Science of the Total Environment*.

[9] Nematollahi S., Ayubi E., Karami M. (2017). Epidemiological characteristics of human brucellosis in Hamadan Province during 2009–2015: results from the national notifiable diseases surveillance system. *International Journal of Infectious Diseases*.

[10] Wu X., Lu Y., Zhou S., Chen L., Xu B. (2016). Impact of climate change on human infectious diseases: empirical evidence and human adaptation. *Environment International*.

[11] He Y., Liu W. J., Jia N., Richardson S., Huang C. (2023). Viral respiratory infections in a rapidly changing climate: the need to prepare for the next pandemic. *EBioMedicine*.

[12] Sun Z.-X., Wang Y., Li Y.-J. (2023). Socioeconomic, meteorological factors and spatiotemporal distribution of human brucellosis in China between 2004 and 2019—a study based on spatial panel model. *PLOS Neglected Tropical Diseases*.

[13] Yang Z. R., Li X., Shao Z. J. (2018). Characteristics on spatial and temporal distribution as well as the driving effect of meteorological factors on brucellosis in Datong city, Shanxi province, 2005-2015. *Zhonghua liu xing bing xue za zhi = Zhonghua liuxingbingxue zazhi*.

[14] Chelghoum A., Boumimez F., Alsamara M. (2023). Asymmetric effects of oil price shocks on the demand for money in Algeria. *The Quarterly Review of Economics and Finance*.

[15] Shin Y., Yu B., Greenwoodnimmo M. (2014). Modelling asymmetric cointegration and dynamic multipliers in a nonlinear ARDL framework. *Festschrift in Honor of Peter Schmidt*.

[16] Bakry W., Nghiem X.-H., Farouk S., Vo X. V. (2023). Does it hurt or help? Revisiting the effects of ICT on economic growth and energy consumption: a nonlinear panel ARDL approach. *Economic Analysis and Policy*.

[17] Zhong Z., Yu S., Wang X. (2013). Human brucellosis in the People’s Republic of China during 2005–2010. *International Journal of Infectious Diseases*.

[18] Clegg L. X., Hankey B. F., Tiwari R., Feuer E. J., Edwards B. K. (2009). Estimating average annual percent change in trend analysis. *Statistics in Medicine*.

[19] Mason C. H., Perreault W. D. (1991). Collinearity, power, and interpretation of multiple regression analysis. *Journal of Marketing Research*.

[20] Zuur A. F., Ieno E. N., Elphick C. S. (2010). A protocol for data exploration to avoid common statistical problems. *Methods in Ecology and Evolution*.

[21] Wang Y., Xu C., Ren J., Li Y., Wu W., Yao S. (2021). Use of meteorological parameters for forecasting scarlet fever morbidity in Tianjin, Northern China. *Environmental Science and Pollution Research*.

[22] Hu W., Li Y., Han W. (2018). Meteorological factors and the incidence of mumps in Fujian province, China, 2005–2013: non-linear effects. *Science of the Total Environment*.

[23] Zheng Y., Zhou M., Wen F. (2021). Asymmetric effects of oil shocks on carbon allowance price: evidence from China. *Energy Economics*.

[24] Musbah H., Aly H. H., Little T. A. (2023). A proposed novel adaptive DC technique for non-stationary data removal. *Heliyon*.

[25] Gaies B., Nakhli M. S., Sahut J. M., Guesmi K. (2021). Is bitcoin rooted in confidence? Unraveling the determinants of globalized digital currencies. *Technological Forecasting and Social Change*.

[26] Raza N., Shahzad S. J. H., Tiwari A. K., Shahbaz M. (2016). Asymmetric impact of gold, oil prices and their volatilities on stock prices of emerging markets. *Resources Policy*.

[27] Diebold F. X., Mariano R. S. (2002). Comparing predictive accuracy. *Journal of Business & Economic Statistics*.

[28] Olfatifar M., Hosseini S. M., Shokri P., Khodakarim S., Khadembashi N., Pordanjani S. R. (2020). How to improve the human brucellosis surveillance system in Kurdistan province, Iran: reduce the delay in the diagnosis time. *Epidemiology and Health*.

[29] Zhang X., Zhang Q., Zhang G., Nie Z., Gui Z., Que H. (2018). A novel hybrid data-driven model for daily land surface temperature forecasting using long short-term memory neural network based on ensemble empirical mode decomposition. *International Journal of Environmental Research and Public Health*.

[30] Islam M. A., Hasan M. N., Tiwari A. (2023). Correlation of dengue and meteorological factors in Bangladesh: a public health concern. *International Journal of Environmental Research and Public Health*.

[31] Vavilala H., Yaladanda N., Kondeti P. K. (2022). Weather integrated malaria prediction system using bayesian structural time series model for northeast states of India. *Environmental Science and Pollution Research*.

[32] Zheng H., Liu D., Zhao X. (2023). Influence and prediction of meteorological factors on brucellosis in a northwest region of China. *Environmental Science and Pollution Research*.

[33] Boubaker H., Larbi O. B. (2022). Dynamic dependence and hedging strategies in BRICS stock markets with oil during crises. *Economic Analysis and Policy*.

[34] Chen H., Lin M.-X., Wang L.-P. (2023). Driving role of climatic and socioenvironmental factors on human brucellosis in China: machine-learning-based predictive analyses. *Infectious Diseases of Poverty*.

[35] Bagheri H., Tapak L., Karami M. (2020). Forecasting the monthly incidence rate of brucellosis in west of Iran using time series and data mining from 2010 to 2019. *PLOS ONE*.

[36] Liang D., Liu D., Yang M. (2021). Spatiotemporal distribution of human brucellosis in Inner Mongolia, China, in 2010–2015, and influencing factors. *Scientific Reports*.

[37] Yang Z., Pang M., Zhou Q. (2020). Spatiotemporal expansion of human brucellosis in Shaanxi province, Northwestern China and model for risk prediction. *PeerJ*.

[38] Xu L., Deng Y. (2022). Spatiotemporal pattern evolution and driving factors of brucellosis in China, 2003–2019. *International Journal of Environmental Research and Public Health*.

[39] Peng R., Wang Y., Zhai J. (2022). Driving effect of multiplex factors on human brucellosis in high incidence region, implication for brucellosis based on one health concept. *One Health*.

[40] Liu K., Yang Z., Liang W., Guo T., Long Y., Shao Z. (2020). Effect of climatic factors on the seasonal fluctuation of human brucellosis in Yulin, Northern China. *BMC Public Health*.

[41] Imai C., Armstrong B., Chalabi Z., Mangtani P., Hashizume M. (2015). Time series regression model for infectious disease and weather. *Environmental Research*.

