# Supplementary material for: Asymmetric Effects of Weather-Integrated Human Brucellosis Forecasting System Using a New Nonlinear Autoregressive Distributed Lag Model
Source: Transbound Emerg Dis. 2024 Mar 5;2024:8381548. doi: 10.1155/2024/8381548 (PMC12017184; doi:10.1155/2024/8381548)
Supplement: Supplementary Materials — Figure S1: geographic snapshot of Henan province. Figure S2: partial autocorrelogram for the differenced HB cases. The presence of local maximum values at 1- and 12-month delays, suggesting the first-order autocorrelation between HB cases. PACF partial autocorrelation function. Figure S3: akaike information criterion (AIC) of top 20 NARDL models. Figure S4: CUSUM graph for the stability test of the NARDL model. The residuals at different time points fell into the 95% confidence intervals, substantiating that the model is stable and valid. CUSUM cumulative sum. Figure S5: akaike information criterion (AIC) of top 20 ARDL models. Table S1: possible NARDL candidates. Table S2: possible ARDL candidates. [file 8381548.f1.docx]

**Supplemental material: Asymmetric effects of weather integrated** **human brucellosis forecasting system using** **a new nonlinear autoregressive distributed lag model**

### Development of NARDL and ARDL models

The dependent and independent variables were shown to be nonstationary based on the ADF test for HB cases (t=-0.47, *P*=0.51), AT (t=-0.03, *P*=0.67), AAP (t=0.70, *P*=0.87), ASH (t=-0.16, *P*=0.63), AWV (t=-0.38, *P*=0.55), AP (t=-0.72, *P*=0.41), and ARH (t=-0.43, *P*=0.53). After processing with a logarithmic transformation or differencing, they became stationary with the t-statistic values being -2.68 (*P*=0.008), -5.00 (*P*<0.001), -4.05 (*P*<0.001), -10.06 (*P*<0.001), -11.19 (*P*<0.001), -10.31 (*P*<0.001), and -9.27 (*P*<0.001). The PACF plot demonstrated that the autocorrelation at a delay of one month were specified (**Figure S2**). Then the bounds test of F=34.29 (which was far greater than the clinical upper bounds [I_0_=1.82, I_1_=2.99]) corroborated the presence of the long-term cointegration asymmetric association between variables. Lastly, a wide range of NARDL models were developed by adjustment for seasonality, autocorrelation, and long-term trend. The NARDL model and optimized NARDL model results were shown in **Tables S1 and Figure S3**. Of the possible candidates, we chose the NARDL(1, 0, 1, 0, 0, 2, 0, 1, 2, 0, 1) specification (in which the lag of Δlog(HB) was one, lags of ΔAT(+) and ΔAT(-) were zero and one, respectively, lags of ΔAP(+) and ΔAP(-) were all zero, lags of ΔASH(+) and ΔASH(-) were two and zero, lags of ΔAWV(+) and ΔAWV(-) were one and two, respectively) as the best possible model because it gave a smaller value of the AIC (2.38), BIC (2.88), and HQ (2.58), along with a lager value of the adjusted *R^2^* (0.35) and log-likehood (-193.62), As shown in **Figure S4**, the CUSUM statistics fell with the 95% confidence intervals, substantiating the stability of the NARDL. Likewise, the ARDL(1, 0, 0, 0, 2, 1) was identified as the best possible specification among the possible ARDL candidates (**Table S2 and Figure S5**).


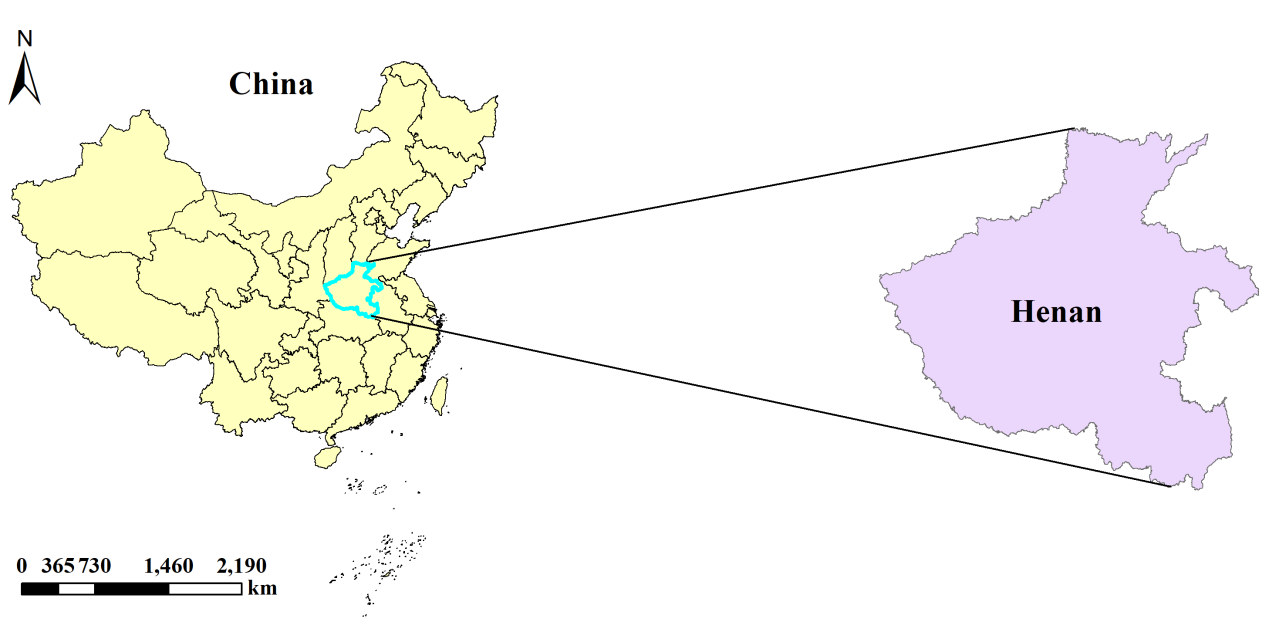


**Figure S1.** Geographic snapshot of Henan province


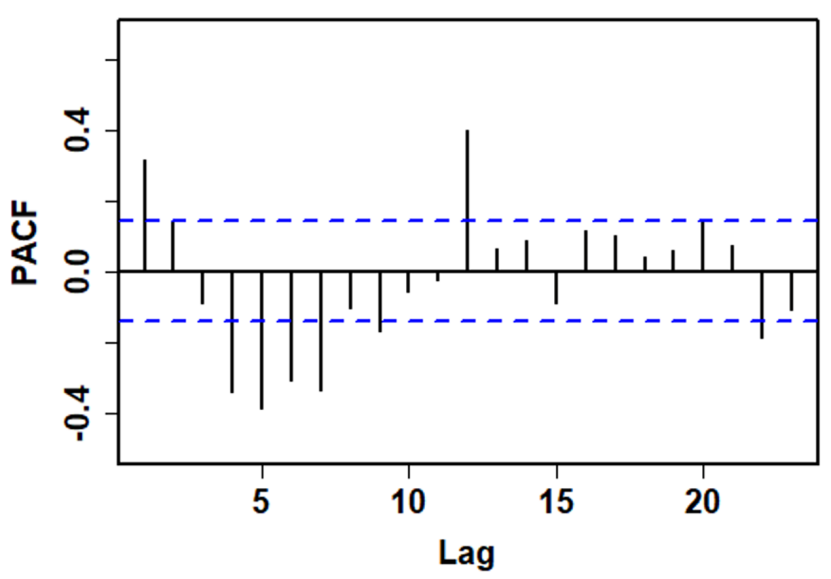


**Figure S2.** Partial autocorrelogram for the differenced HB cases. The presence of local maximum values at 1- and 12-month delays, suggesting the first-order autocorrelation between HB cases. PACF partial autocorrelation function.

**Figure S3.** Akaike information criterion (AIC) of top 20 NARDL models


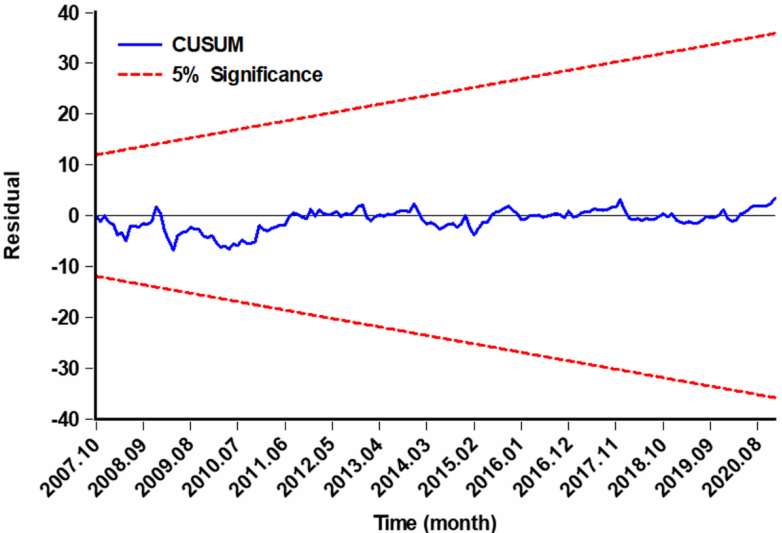


**Figure S4.** CUSUM graph for the stability test of the NARDL model. The residuals at different time points fell into the 95% confidence intervals, substantiating that the model is stable and valid. CUSUM cumulative sum.

**Figure S5.** Akaike information criterion (AIC) of top 20 ARDL models

**Table S1.** Possible NARDL candidates

| Model | LogL | AIC* | BIC | HQ | Adj R^2^ | Specification |
| --- | --- | --- | --- | --- | --- | --- |
| 1 | -193.616 | 2.380917 | 2.881998 | 2.583955 | 0.346006 | ARDL(1, 0, 1, 0, 0, 2, 0, 1, 2, 0, 1) |
| 2 | -193.641 | 2.381192 | 2.882273 | 2.584230 | 0.345826 | ARDL(1, 0, 1, 0, 0, 1, 1, 1, 2, 0, 1) |
| 3 | -194.882 | 2.383762 | 2.867564 | 2.579798 | 0.341260 | ARDL(1, 0, 1, 0, 0, 1, 0, 1, 2, 0, 1) |
| 4 | -192.904 | 2.383996 | 2.902355 | 2.594035 | 0.346834 | ARDL(1, 2, 1, 0, 0, 1, 0, 1, 2, 0, 1) |
| 5 | -194.906 | 2.384023 | 2.867825 | 2.580060 | 0.341088 | ARDL(1, 0, 1, 0, 0, 0, 0, 1, 2, 1, 1) |
| 6 | -195.952 | 2.384511 | 2.851034 | 2.573546 | 0.337842 | ARDL(1, 0, 1, 0, 0, 0, 0, 1, 2, 0, 1) |
| 7 | -191.984 | 2.384858 | 2.920496 | 2.601899 | 0.349079 | ARDL(1, 2, 1, 0, 1, 1, 0, 1, 2, 0, 1) |
| 8 | -192.985 | 2.384871 | 2.903230 | 2.594910 | 0.346262 | ARDL(1, 0, 1, 0, 1, 2, 0, 1, 2, 0, 1) |
| 9 | -194.016 | 2.385201 | 2.886282 | 2.588239 | 0.343198 | ARDL(1, 0, 1, 1, 1, 0, 0, 1, 2, 0, 1) |
| 10 | -194.053 | 2.385589 | 2.886670 | 2.588627 | 0.342943 | ARDL(1, 2, 1, 0, 0, 0, 0, 1, 2, 0, 1) |
| 11 | -194.077 | 2.385848 | 2.886929 | 2.588886 | 0.342773 | ARDL(1, 0, 1, 0, 0, 1, 0, 1, 2, 1, 1) |
| 12 | -194.129 | 2.386409 | 2.887490 | 2.589447 | 0.342404 | ARDL(1, 0, 1, 0, 1, 1, 0, 1, 2, 0, 1) |
| 13 | -193.136 | 2.386480 | 2.904840 | 2.596520 | 0.345209 | ARDL(1, 0, 1, 0, 1, 1, 1, 1, 2, 0, 1) |
| 14 | -195.143 | 2.386553 | 2.870355 | 2.582590 | 0.339418 | ARDL(1, 0, 1, 1, 0, 0, 0, 1, 2, 0, 1) |
| 15 | -194.153 | 2.386664 | 2.887745 | 2.589702 | 0.342237 | ARDL(1, 0, 1, 0, 2, 0, 0, 1, 2, 0, 1) |
| 16 | -193.172 | 2.386867 | 2.905227 | 2.596907 | 0.344956 | ARDL(1, 0, 1, 1, 1, 1, 0, 1, 2, 0, 1) |
| 17 | -193.192 | 2.387082 | 2.905442 | 2.597122 | 0.344815 | ARDL(1, 0, 1, 0, 2, 1, 0, 1, 2, 0, 1) |
| 18 | -193.205 | 2.387219 | 2.905578 | 2.597258 | 0.344725 | ARDL(1, 2, 1, 0, 1, 0, 0, 1, 2, 0, 1) |
| 19 | -195.207 | 2.387243 | 2.871045 | 2.583280 | 0.338962 | ARDL(1, 0, 1, 0, 0, 0, 0, 1, 2, 0, 2) |
| 20 | -193.228 | 2.387463 | 2.905823 | 2.597502 | 0.344565 | ARDL(1, 0, 2, 0, 0, 2, 0, 1, 2, 0, 1) |
| 21 | -193.243 | 2.387630 | 2.905990 | 2.597669 | 0.344456 | ARDL(1, 0, 2, 0, 0, 1, 1, 1, 2, 0, 1) |
| 22 | -194.256 | 2.387762 | 2.888843 | 2.590800 | 0.341514 | ARDL(1, 0, 1, 0, 1, 0, 0, 1, 2, 1, 1) |
| 23 | -195.264 | 2.387855 | 2.871658 | 2.583892 | 0.338558 | ARDL(1, 0, 1, 0, 1, 0, 0, 1, 2, 0, 1) |
| 24 | -193.289 | 2.388115 | 2.906475 | 2.598155 | 0.344138 | ARDL(1, 0, 1, 0, 0, 1, 1, 2, 2, 0, 1) |
| 25 | -191.291 | 2.388137 | 2.941054 | 2.612179 | 0.349720 | ARDL(1, 2, 1, 0, 2, 1, 0, 1, 2, 0, 1) |
| 26 | -192.293 | 2.388156 | 2.923794 | 2.605197 | 0.346929 | ARDL(1, 2, 1, 0, 0, 1, 1, 1, 2, 0, 1) |
| 27 | -194.297 | 2.388205 | 2.889286 | 2.591243 | 0.341222 | ARDL(1, 0, 1, 0, 0, 1, 0, 1, 2, 0, 2) |
| 28 | -192.302 | 2.388253 | 2.923892 | 2.605294 | 0.346865 | ARDL(1, 2, 1, 1, 1, 0, 0, 1, 2, 0, 1) |
| 29 | -194.306 | 2.388303 | 2.889384 | 2.591342 | 0.341157 | ARDL(1, 0, 1, 1, 0, 1, 0, 1, 2, 0, 1) |
| 30 | -192.31 | 2.388343 | 2.923982 | 2.605384 | 0.346806 | ARDL(1, 2, 1, 0, 0, 2, 0, 1, 2, 0, 1) |
| 31 | -191.337 | 2.388633 | 2.941550 | 2.612675 | 0.349397 | ARDL(1, 2, 1, 1, 1, 1, 0, 1, 2, 0, 1) |
| 32 | -192.34 | 2.388668 | 2.924306 | 2.605709 | 0.346594 | ARDL(1, 2, 1, 0, 0, 1, 0, 2, 2, 0, 1) |
| 33 | -192.354 | 2.388812 | 2.924450 | 2.605853 | 0.346500 | ARDL(1, 2, 1, 0, 2, 0, 0, 1, 2, 0, 1) |
| 34 | -193.366 | 2.388936 | 2.907296 | 2.598975 | 0.343599 | ARDL(1, 0, 1, 0, 1, 1, 0, 1, 2, 1, 1) |
| 35 | -193.368 | 2.388960 | 2.907320 | 2.599000 | 0.343583 | ARDL(1, 0, 1, 0, 0, 2, 0, 2, 2, 0, 1) |
| 36 | -193.378 | 2.389071 | 2.907431 | 2.599111 | 0.343510 | ARDL(1, 0, 1, 0, 0, 2, 0, 1, 2, 1, 1) |
| 37 | -193.405 | 2.389354 | 2.907714 | 2.599394 | 0.343324 | ARDL(1, 0, 1, 0, 0, 2, 1, 1, 2, 0, 1) |
| 38 | -193.421 | 2.389529 | 2.907889 | 2.599569 | 0.343210 | ARDL(1, 0, 1, 1, 0, 1, 1, 1, 2, 0, 1) |
| 39 | -193.426 | 2.389580 | 2.907940 | 2.599620 | 0.343176 | ARDL(1, 0, 1, 1, 0, 2, 0, 1, 2, 0, 1) |
| 40 | -195.427 | 2.389596 | 2.873399 | 2.585633 | 0.337405 | ARDL(1, 0, 1, 0, 0, 0, 1, 1, 2, 0, 1) |
| 41 | -193.439 | 2.389726 | 2.908086 | 2.599766 | 0.343080 | ARDL(1, 2, 1, 0, 0, 0, 0, 1, 2, 1, 1) |
| 42 | -191.444 | 2.389780 | 2.942697 | 2.613822 | 0.348651 | ARDL(1, 2, 1, 0, 0, 1, 1, 2, 2, 0, 1) |
| 43 | -194.486 | 2.390223 | 2.891304 | 2.593261 | 0.339891 | ARDL(1, 0, 1, 0, 1, 0, 0, 1, 2, 0, 2) |
| 44 | -192.497 | 2.390344 | 2.925983 | 2.607385 | 0.345498 | ARDL(1, 2, 1, 0, 0, 1, 0, 1, 2, 1, 1) |
| 45 | -193.513 | 2.390511 | 2.908870 | 2.600550 | 0.342565 | ARDL(1, 0, 1, 0, 2, 0, 0, 1, 2, 1, 1) |
| 46 | -191.514 | 2.390525 | 2.943442 | 2.614567 | 0.348165 | ARDL(1, 2, 1, 0, 1, 2, 0, 1, 2, 0, 1) |
| 47 | -193.518 | 2.390566 | 2.908925 | 2.600605 | 0.342528 | ARDL(1, 0, 1, 0, 1, 1, 0, 1, 2, 0, 2) |
| 48 | -193.525 | 2.390641 | 2.909001 | 2.600681 | 0.342479 | ARDL(1, 2, 1, 1, 0, 0, 0, 1, 2, 0, 1) |
| 49 | -192.546 | 2.390864 | 2.926502 | 2.607905 | 0.345158 | ARDL(1, 0, 1, 1, 1, 2, 0, 1, 2, 0, 1) |
| 50 | -191.546 | 2.390867 | 2.943784 | 2.614910 | 0.347942 | ARDL(1, 2, 1, 0, 1, 1, 0, 2, 2, 0, 1) |
| 51 | -193.558 | 2.390999 | 2.909358 | 2.601038 | 0.342244 | ARDL(1, 0, 1, 0, 0, 2, 0, 1, 3, 0, 1) |
| 52 | -192.563 | 2.391048 | 2.926687 | 2.608089 | 0.345037 | ARDL(1, 0, 1, 0, 2, 2, 0, 1, 2, 0, 1) |
| 53 | -193.563 | 2.391049 | 2.909408 | 2.601088 | 0.342211 | ARDL(1, 0, 1, 0, 0, 1, 1, 1, 2, 0, 2) |
| 54 | -193.564 | 2.391058 | 2.909418 | 2.601098 | 0.342205 | ARDL(1, 0, 1, 0, 0, 1, 1, 1, 2, 1, 1) |
| 55 | -193.567 | 2.391096 | 2.909456 | 2.601136 | 0.342180 | ARDL(1, 1, 1, 0, 0, 2, 0, 1, 2, 0, 1) |
| 56 | -192.569 | 2.391111 | 2.926749 | 2.608152 | 0.344996 | ARDL(1, 2, 1, 1, 0, 1, 0, 1, 2, 0, 1) |
| 57 | -192.578 | 2.391204 | 2.926842 | 2.608244 | 0.344935 | ARDL(1, 0, 1, 0, 2, 1, 1, 1, 2, 0, 1) |
| 58 | -191.588 | 2.391317 | 2.944234 | 2.615359 | 0.347649 | ARDL(1, 2, 1, 0, 1, 1, 1, 1, 2, 0, 1) |
| 59 | -191.592 | 2.391353 | 2.944270 | 2.615395 | 0.347625 | ARDL(1, 2, 1, 0, 0, 2, 0, 2, 2, 0, 1) |
| 60 | -193.596 | 2.391403 | 2.909763 | 2.601442 | 0.341978 | ARDL(1, 0, 1, 0, 0, 1, 2, 1, 2, 0, 1) |
| 61 | -194.615 | 2.391609 | 2.892690 | 2.594647 | 0.338976 | ARDL(1, 0, 1, 1, 0, 0, 0, 1, 2, 1, 1) |
| 62 | -193.616 | 2.391611 | 2.909970 | 2.601650 | 0.341841 | ARDL(1, 0, 1, 0, 0, 2, 0, 1, 2, 0, 2) |
| 63 | -193.616 | 2.391612 | 2.909972 | 2.601652 | 0.341840 | ARDL(1, 0, 1, 0, 0, 3, 0, 1, 2, 0, 1) |
| 64 | -194.616 | 2.391613 | 2.892694 | 2.594651 | 0.338973 | ARDL(1, 0, 1, 2, 0, 0, 0, 1, 2, 0, 1) |
| 65 | -193.618 | 2.391641 | 2.910001 | 2.601681 | 0.341821 | ARDL(1, 0, 1, 0, 0, 1, 1, 1, 3, 0, 1) |
| 66 | -191.621 | 2.391667 | 2.944584 | 2.615709 | 0.347420 | ARDL(1, 2, 1, 0, 1, 1, 0, 1, 2, 1, 1) |
| 67 | -192.621 | 2.391671 | 2.927309 | 2.608712 | 0.344629 | ARDL(1, 0, 2, 0, 1, 2, 0, 1, 2, 0, 1) |
| 68 | -193.628 | 2.391738 | 2.910098 | 2.601778 | 0.341757 | ARDL(1, 1, 1, 0, 0, 1, 1, 1, 2, 0, 1) |
| 69 | -192.635 | 2.391823 | 2.927462 | 2.608864 | 0.344529 | ARDL(1, 2, 1, 0, 1, 0, 0, 1, 2, 1, 1) |
| 70 | -192.652 | 2.392001 | 2.927640 | 2.609042 | 0.344413 | ARDL(1, 0, 1, 1, 1, 1, 1, 1, 2, 0, 1) |
| 71 | -193.659 | 2.392074 | 2.910434 | 2.602114 | 0.341536 | ARDL(1, 0, 1, 1, 1, 0, 0, 1, 2, 1, 1) |
| 72 | -194.673 | 2.392223 | 2.893304 | 2.595262 | 0.338569 | ARDL(1, 0, 2, 0, 0, 1, 0, 1, 2, 0, 1) |
| 73 | -192.685 | 2.392350 | 2.927989 | 2.609391 | 0.344184 | ARDL(1, 2, 1, 0, 0, 1, 0, 1, 2, 0, 2) |
| 74 | -192.694 | 2.392449 | 2.928088 | 2.609490 | 0.344119 | ARDL(1, 3, 1, 0, 0, 1, 0, 1, 2, 0, 1) |
| 75 | -197.696 | 2.392472 | 2.841717 | 2.574507 | 0.329563 | ARDL(1, 0, 1, 0, 0, 0, 0, 1, 1, 0, 1) |
| 76 | -192.713 | 2.392649 | 2.928287 | 2.609690 | 0.343988 | ARDL(1, 0, 1, 0, 2, 1, 0, 1, 2, 1, 1) |
| 77 | -193.722 | 2.392748 | 2.911107 | 2.602787 | 0.341092 | ARDL(1, 2, 1, 0, 0, 0, 0, 1, 2, 0, 2) |
| 78 | -191.74 | 2.392940 | 2.945857 | 2.616983 | 0.346589 | ARDL(1, 3, 1, 0, 1, 1, 0, 1, 2, 0, 1) |
| 79 | -194.745 | 2.392992 | 2.894073 | 2.596030 | 0.338061 | ARDL(1, 0, 1, 0, 0, 0, 0, 1, 2, 2, 1) |
| 80 | -194.748 | 2.393027 | 2.894108 | 2.596065 | 0.338038 | ARDL(1, 0, 1, 0, 0, 0, 0, 1, 2, 0, 3) |
| 81 | -192.748 | 2.393028 | 2.928667 | 2.610069 | 0.343739 | ARDL(1, 0, 1, 0, 1, 2, 0, 1, 2, 1, 1) |
| 82 | -193.754 | 2.393096 | 2.911455 | 2.603135 | 0.340863 | ARDL(1, 0, 1, 2, 0, 1, 0, 1, 2, 0, 1) |
| 83 | -191.763 | 2.393187 | 2.946104 | 2.617229 | 0.346428 | ARDL(1, 2, 1, 0, 1, 1, 0, 1, 2, 0, 2) |
| 84 | -192.771 | 2.393268 | 2.928906 | 2.610309 | 0.343582 | ARDL(1, 0, 2, 0, 1, 1, 1, 1, 2, 0, 1) |
| 85 | -193.775 | 2.393314 | 2.911673 | 2.603353 | 0.340719 | ARDL(1, 2, 1, 0, 0, 0, 0, 2, 2, 0, 1) |
| 86 | -194.8 | 2.393585 | 2.894666 | 2.596623 | 0.337668 | ARDL(1, 0, 1, 0, 0, 1, 0, 2, 2, 0, 1) |
| 87 | -192.8 | 2.393585 | 2.929224 | 2.610626 | 0.343373 | ARDL(1, 0, 3, 0, 0, 1, 1, 1, 2, 0, 1) |
| 88 | -190.805 | 2.393640 | 2.963836 | 2.624684 | 0.348887 | ARDL(1, 2, 1, 0, 2, 1, 0, 2, 2, 0, 1) |
| 89 | -194.819 | 2.393782 | 2.894863 | 2.596820 | 0.337538 | ARDL(1, 0, 1, 0, 0, 0, 0, 1, 2, 1, 2) |
| 90 | -192.819 | 2.393787 | 2.929425 | 2.610828 | 0.343241 | ARDL(1, 0, 1, 0, 1, 2, 0, 2, 2, 0, 1) |
| 91 | -198.825 | 2.393848 | 2.825814 | 2.568881 | 0.325610 | ARDL(1, 0, 1, 0, 0, 0, 0, 0, 1, 0, 1) |
| 92 | -193.828 | 2.393878 | 2.912238 | 2.603918 | 0.340347 | ARDL(1, 0, 1, 1, 1, 0, 0, 1, 2, 0, 2) |
| 93 | -194.829 | 2.393897 | 2.894978 | 2.596935 | 0.337462 | ARDL(1, 0, 2, 0, 0, 0, 0, 1, 2, 1, 1) |
| 94 | -194.83 | 2.393901 | 2.894982 | 2.596939 | 0.337459 | ARDL(1, 0, 1, 0, 0, 0, 0, 2, 2, 1, 1) |
| 95 | -190.833 | 2.393937 | 2.964133 | 2.624980 | 0.348693 | ARDL(1, 2, 1, 1, 1, 1, 0, 2, 2, 0, 1) |
| 96 | -197.833 | 2.393938 | 2.843183 | 2.575972 | 0.328579 | ARDL(1, 0, 1, 0, 0, 1, 0, 0, 1, 0, 1) |
| 97 | -193.838 | 2.393988 | 2.912348 | 2.604028 | 0.340274 | ARDL(1, 0, 2, 0, 0, 1, 0, 1, 2, 1, 1) |
| 98 | -192.841 | 2.394017 | 2.929656 | 2.611058 | 0.343090 | ARDL(1, 2, 1, 0, 0, 1, 0, 1, 3, 0, 1) |
| 99 | -194.845 | 2.394066 | 2.895147 | 2.597104 | 0.337350 | ARDL(1, 0, 1, 1, 0, 0, 1, 1, 2, 0, 1) |
| 100 | -194.847 | 2.394086 | 2.895168 | 2.597125 | 0.337336 | ARDL(1, 1, 1, 0, 0, 1, 0, 1, 2, 0, 1) |
| 101 | -191.85 | 2.394114 | 2.947031 | 2.618156 | 0.345822 | ARDL(1, 2, 1, 0, 0, 1, 0, 2, 2, 1, 1) |
| 102 | -194.86 | 2.394220 | 2.895301 | 2.597258 | 0.337248 | ARDL(1, 0, 1, 0, 0, 0, 1, 1, 2, 1, 1) |
| 103 | -192.862 | 2.394247 | 2.929886 | 2.611288 | 0.342938 | ARDL(1, 0, 1, 0, 1, 2, 1, 1, 2, 0, 1) |
| 104 | -194.868 | 2.394305 | 2.895386 | 2.597343 | 0.337191 | ARDL(1, 0, 1, 0, 0, 1, 0, 1, 3, 0, 1) |
| 105 | -192.868 | 2.394306 | 2.929945 | 2.611347 | 0.342900 | ARDL(1, 2, 1, 0, 1, 0, 0, 1, 2, 0, 2) |
| 106 | -194.868 | 2.394310 | 2.895391 | 2.597348 | 0.337188 | ARDL(1, 0, 1, 1, 0, 0, 0, 1, 2, 0, 2) |
| 107 | -193.87 | 2.394331 | 2.912691 | 2.604370 | 0.340048 | ARDL(1, 3, 1, 0, 0, 0, 0, 1, 2, 0, 1) |
| 108 | -193.876 | 2.394393 | 2.912753 | 2.604432 | 0.340007 | ARDL(1, 0, 1, 1, 0, 1, 0, 1, 2, 1, 1) |
| 109 | -192.889 | 2.394531 | 2.930170 | 2.611572 | 0.342752 | ARDL(1, 2, 2, 0, 0, 1, 0, 1, 2, 0, 1) |
| 110 | -192.891 | 2.394561 | 2.930200 | 2.611602 | 0.342732 | ARDL(1, 0, 1, 0, 1, 1, 1, 2, 2, 0, 1) |
| 111 | -193.892 | 2.394570 | 2.912929 | 2.604609 | 0.339891 | ARDL(1, 0, 1, 0, 0, 1, 0, 1, 2, 0, 3) |
| 112 | -194.893 | 2.394580 | 2.895661 | 2.597618 | 0.337009 | ARDL(1, 0, 1, 0, 0, 0, 0, 1, 3, 1, 1) |
| 113 | -192.895 | 2.394601 | 2.930240 | 2.611642 | 0.342706 | ARDL(1, 0, 1, 1, 1, 1, 0, 1, 2, 1, 1) |
| 114 | -193.895 | 2.394603 | 2.912962 | 2.604642 | 0.339869 | ARDL(1, 2, 1, 0, 0, 0, 1, 1, 2, 0, 1) |
| 115 | -194.898 | 2.394627 | 2.895708 | 2.597665 | 0.336978 | ARDL(1, 0, 1, 0, 1, 0, 1, 1, 2, 0, 1) |
| 116 | -193.902 | 2.394672 | 2.913032 | 2.604711 | 0.339823 | ARDL(1, 0, 1, 0, 0, 1, 0, 2, 2, 1, 1) |
| 117 | -192.903 | 2.394683 | 2.930321 | 2.611724 | 0.342652 | ARDL(1, 0, 1, 2, 0, 1, 1, 1, 2, 0, 1) |
| 118 | -194.906 | 2.394715 | 2.895796 | 2.597753 | 0.336919 | ARDL(1, 1, 1, 0, 0, 0, 0, 1, 2, 1, 1) |
| 119 | -195.907 | 2.394724 | 2.878527 | 2.590761 | 0.333998 | ARDL(1, 0, 2, 0, 0, 0, 0, 1, 2, 0, 1) |
| 120 | -193.907 | 2.394731 | 2.913091 | 2.604771 | 0.339784 | ARDL(1, 0, 1, 1, 1, 0, 1, 1, 2, 0, 1) |
| 121 | -190.921 | 2.394877 | 2.965073 | 2.625921 | 0.348081 | ARDL(1, 2, 1, 0, 1, 1, 1, 2, 2, 0, 1) |
| 122 | -193.925 | 2.394920 | 2.913279 | 2.604959 | 0.339660 | ARDL(1, 0, 1, 0, 2, 0, 0, 1, 2, 0, 2) |
| 123 | -192.927 | 2.394937 | 2.930575 | 2.611977 | 0.342485 | ARDL(1, 0, 2, 0, 2, 1, 0, 1, 2, 0, 1) |
| 124 | -193.932 | 2.394994 | 2.913354 | 2.605034 | 0.339610 | ARDL(1, 0, 2, 0, 1, 1, 0, 1, 2, 0, 1) |
| 125 | -195.932 | 2.395000 | 2.878802 | 2.591036 | 0.333815 | ARDL(1, 0, 1, 0, 0, 0, 0, 1, 3, 0, 1) |
| 126 | -191.933 | 2.395004 | 2.947921 | 2.619046 | 0.345239 | ARDL(1, 2, 1, 1, 0, 1, 0, 2, 2, 0, 1) |
| 127 | -192.935 | 2.395032 | 2.930670 | 2.612073 | 0.342423 | ARDL(1, 0, 2, 0, 0, 1, 1, 2, 2, 0, 1) |
| 128 | -193.937 | 2.395053 | 2.913412 | 2.605092 | 0.339572 | ARDL(1, 0, 1, 1, 2, 0, 0, 1, 2, 0, 1) |
| 129 | -195.94 | 2.395079 | 2.878882 | 2.591116 | 0.333762 | ARDL(1, 1, 1, 0, 0, 0, 0, 1, 2, 0, 1) |
| 130 | -195.94 | 2.395082 | 2.878885 | 2.591119 | 0.333760 | ARDL(1, 0, 1, 0, 0, 0, 0, 2, 2, 0, 1) |
| 131 | -190.942 | 2.395105 | 2.965301 | 2.626148 | 0.347932 | ARDL(1, 2, 1, 0, 1, 2, 0, 2, 2, 0, 1) |
| 132 | -192.95 | 2.395188 | 2.930827 | 2.612229 | 0.342320 | ARDL(1, 0, 1, 0, 0, 1, 1, 1, 2, 0, 3) |
| 133 | -192.95 | 2.395192 | 2.930830 | 2.612233 | 0.342318 | ARDL(1, 1, 1, 0, 1, 2, 0, 1, 2, 0, 1) |
| 134 | -193.951 | 2.395201 | 2.913560 | 2.605240 | 0.339474 | ARDL(1, 0, 2, 1, 1, 0, 0, 1, 2, 0, 1) |
| 135 | -192.953 | 2.395219 | 2.930857 | 2.612260 | 0.342300 | ARDL(1, 0, 2, 1, 1, 1, 0, 1, 2, 0, 1) |
| 136 | -193.956 | 2.395251 | 2.913611 | 2.605291 | 0.339441 | ARDL(1, 0, 1, 0, 1, 0, 0, 1, 2, 0, 3) |
| 137 | -196.959 | 2.395280 | 2.861804 | 2.584315 | 0.330673 | ARDL(1, 0, 0, 0, 0, 1, 0, 1, 2, 0, 1) |
| 138 | -191.959 | 2.395284 | 2.948201 | 2.619326 | 0.345056 | ARDL(1, 2, 1, 0, 1, 1, 0, 1, 3, 0, 1) |
| 139 | -193.961 | 2.395309 | 2.913668 | 2.605348 | 0.339403 | ARDL(1, 0, 3, 0, 0, 1, 0, 1, 2, 0, 1) |
| 140 | -192.963 | 2.395328 | 2.930966 | 2.612369 | 0.342228 | ARDL(1, 0, 1, 0, 1, 2, 0, 1, 3, 0, 1) |
| 141 | -193.968 | 2.395380 | 2.913740 | 2.605420 | 0.339356 | ARDL(1, 0, 1, 0, 0, 1, 0, 1, 2, 2, 1) |
| 142 | -191.972 | 2.395420 | 2.948337 | 2.619463 | 0.344966 | ARDL(1, 2, 2, 0, 1, 1, 0, 1, 2, 0, 1) |
| 143 | -192.972 | 2.395425 | 2.931064 | 2.612466 | 0.342164 | ARDL(1, 0, 2, 0, 0, 2, 1, 1, 2, 0, 1) |
| 144 | -193.975 | 2.395460 | 2.913819 | 2.605499 | 0.339303 | ARDL(1, 2, 1, 0, 0, 0, 0, 1, 3, 0, 1) |
| 145 | -192.982 | 2.395527 | 2.931166 | 2.612568 | 0.342097 | ARDL(1, 0, 1, 0, 1, 2, 0, 1, 2, 0, 2) |
| 146 | -192.985 | 2.395559 | 2.931198 | 2.612600 | 0.342076 | ARDL(1, 0, 1, 0, 1, 3, 0, 1, 2, 0, 1) |
| 147 | -192.987 | 2.395582 | 2.931221 | 2.612623 | 0.342061 | ARDL(1, 0, 3, 0, 1, 1, 0, 1, 2, 0, 1) |
| 148 | -196.988 | 2.395597 | 2.862121 | 2.584633 | 0.330460 | ARDL(1, 0, 1, 0, 0, 1, 0, 1, 1, 0, 1) |
| 149 | -192.992 | 2.395637 | 2.931276 | 2.612678 | 0.342025 | ARDL(1, 3, 1, 0, 1, 0, 0, 1, 2, 0, 1) |
| 150 | -193.994 | 2.395655 | 2.914015 | 2.605695 | 0.339174 | ARDL(1, 0, 1, 0, 2, 0, 1, 1, 2, 0, 1) |
| 151 | -193.995 | 2.395667 | 2.914027 | 2.605707 | 0.339166 | ARDL(1, 0, 1, 2, 1, 0, 0, 1, 2, 0, 1) |
| 152 | -193.996 | 2.395676 | 2.914036 | 2.605715 | 0.339160 | ARDL(1, 1, 1, 1, 1, 0, 0, 1, 2, 0, 1) |
| 153 | -197.998 | 2.395702 | 2.844947 | 2.577736 | 0.327394 | ARDL(1, 0, 0, 0, 0, 0, 0, 1, 2, 0, 1) |
| 154 | -193.998 | 2.395705 | 2.914064 | 2.605744 | 0.339141 | ARDL(1, 0, 1, 1, 1, 0, 0, 2, 2, 0, 1) |
| 155 | -195.002 | 2.395742 | 2.896823 | 2.598780 | 0.336238 | ARDL(1, 0, 1, 0, 0, 0, 2, 1, 2, 0, 1) |
| 156 | -194.002 | 2.395746 | 2.914105 | 2.605785 | 0.339114 | ARDL(1, 0, 1, 0, 0, 1, 0, 1, 2, 1, 2) |
| 157 | -193.004 | 2.395768 | 2.931407 | 2.612809 | 0.341938 | ARDL(1, 0, 2, 0, 0, 2, 0, 1, 2, 1, 1) |
| 158 | -197.006 | 2.395784 | 2.862307 | 2.584819 | 0.330336 | ARDL(1, 0, 1, 0, 1, 0, 0, 1, 1, 0, 1) |
| 159 | -193.011 | 2.395840 | 2.931479 | 2.612881 | 0.341891 | ARDL(1, 0, 1, 0, 1, 1, 1, 1, 2, 0, 2) |
| 160 | -193.014 | 2.395874 | 2.931512 | 2.612915 | 0.341869 | ARDL(1, 2, 1, 0, 1, 0, 0, 2, 2, 0, 1) |
| 161 | -193.014 | 2.395877 | 2.931515 | 2.612917 | 0.341867 | ARDL(1, 0, 1, 1, 1, 1, 0, 1, 2, 0, 2) |
| 162 | -194.015 | 2.395881 | 2.914241 | 2.605921 | 0.339024 | ARDL(1, 0, 1, 2, 0, 0, 0, 1, 2, 1, 1) |
| 163 | -194.016 | 2.395894 | 2.914254 | 2.605934 | 0.339016 | ARDL(1, 0, 1, 1, 1, 0, 0, 1, 3, 0, 1) |
| 164 | -192.017 | 2.395906 | 2.948823 | 2.619948 | 0.344648 | ARDL(1, 2, 1, 0, 2, 0, 0, 1, 2, 1, 1) |
| 165 | -193.023 | 2.395969 | 2.931607 | 2.613010 | 0.341806 | ARDL(1, 0, 1, 0, 2, 1, 0, 1, 2, 0, 2) |
| 166 | -193.023 | 2.395970 | 2.931608 | 2.613010 | 0.341806 | ARDL(1, 0, 1, 0, 1, 1, 1, 1, 2, 1, 1) |
| 167 | -193.023 | 2.395972 | 2.931610 | 2.613013 | 0.341804 | ARDL(1, 0, 2, 0, 0, 2, 0, 2, 2, 0, 1) |
| 168 | -194.025 | 2.395989 | 2.914349 | 2.606029 | 0.338953 | ARDL(1, 0, 1, 0, 1, 0, 0, 1, 2, 2, 1) |
| 169 | -193.032 | 2.396060 | 2.931699 | 2.613101 | 0.341746 | ARDL(1, 0, 2, 1, 0, 1, 1, 1, 2, 0, 1) |
| 170 | -192.034 | 2.396081 | 2.948998 | 2.620123 | 0.344534 | ARDL(1, 2, 1, 1, 1, 0, 0, 2, 2, 0, 1) |
| 171 | -192.035 | 2.396100 | 2.949017 | 2.620142 | 0.344521 | ARDL(1, 3, 1, 0, 0, 1, 0, 2, 2, 0, 1) |
| 172 | -195.04 | 2.396146 | 2.897227 | 2.599184 | 0.335970 | ARDL(1, 0, 1, 0, 0, 0, 1, 1, 2, 0, 2) |
| 173 | -194.04 | 2.396146 | 2.914506 | 2.606186 | 0.338849 | ARDL(1, 2, 2, 0, 0, 0, 0, 1, 2, 0, 1) |
| 174 | -193.046 | 2.396211 | 2.931849 | 2.613252 | 0.341647 | ARDL(1, 0, 1, 0, 1, 1, 0, 1, 2, 0, 3) |
| 175 | -193.047 | 2.396223 | 2.931862 | 2.613264 | 0.341639 | ARDL(1, 0, 3, 0, 0, 2, 0, 1, 2, 0, 1) |
| 176 | -193.047 | 2.396224 | 2.931862 | 2.613265 | 0.341639 | ARDL(1, 0, 2, 1, 0, 2, 0, 1, 2, 0, 1) |
| 177 | -193.047 | 2.396228 | 2.931866 | 2.613269 | 0.341636 | ARDL(1, 2, 1, 2, 0, 0, 0, 1, 2, 0, 1) |
| 178 | -193.049 | 2.396248 | 2.931887 | 2.613289 | 0.341622 | ARDL(1, 0, 1, 1, 0, 1, 1, 2, 2, 0, 1) |
| 179 | -193.051 | 2.396267 | 2.931905 | 2.613308 | 0.341610 | ARDL(1, 0, 1, 0, 0, 2, 1, 2, 2, 0, 1) |
| 180 | -197.055 | 2.396310 | 2.862833 | 2.585345 | 0.329983 | ARDL(1, 0, 1, 0, 1, 1, 0, 0, 1, 0, 1) |
| 181 | -197.06 | 2.396367 | 2.862891 | 2.585403 | 0.329945 | ARDL(1, 0, 1, 0, 0, 0, 0, 1, 1, 1, 1) |
| 182 | -193.063 | 2.396393 | 2.932032 | 2.613434 | 0.341527 | ARDL(1, 2, 1, 0, 0, 0, 0, 2, 2, 1, 1) |
| 183 | -194.065 | 2.396417 | 2.914776 | 2.606456 | 0.338670 | ARDL(1, 0, 2, 0, 0, 1, 0, 1, 2, 0, 2) |
| 184 | -194.067 | 2.396439 | 2.914798 | 2.606478 | 0.338656 | ARDL(1, 0, 1, 0, 0, 1, 0, 1, 3, 1, 1) |
| 185 | -192.067 | 2.396440 | 2.949357 | 2.620483 | 0.344298 | ARDL(1, 2, 1, 2, 0, 1, 0, 1, 2, 0, 1) |
| 186 | -194.068 | 2.396446 | 2.914806 | 2.606485 | 0.338651 | ARDL(1, 1, 1, 0, 0, 1, 0, 1, 2, 1, 1) |
| 187 | -194.069 | 2.396457 | 2.914817 | 2.606497 | 0.338644 | ARDL(1, 0, 1, 1, 0, 1, 0, 1, 2, 0, 2) |
| 188 | -193.07 | 2.396466 | 2.932105 | 2.613507 | 0.341479 | ARDL(1, 0, 1, 1, 2, 1, 0, 1, 2, 0, 1) |
| 189 | -194.07 | 2.396469 | 2.914829 | 2.606509 | 0.338636 | ARDL(1, 0, 2, 0, 2, 0, 0, 1, 2, 0, 1) |
| 190 | -195.072 | 2.396495 | 2.897576 | 2.599533 | 0.335738 | ARDL(1, 0, 2, 1, 0, 0, 0, 1, 2, 0, 1) |
| 191 | -192.073 | 2.396504 | 2.949421 | 2.620546 | 0.344256 | ARDL(1, 3, 1, 0, 0, 1, 1, 1, 2, 0, 1) |
| 192 | -191.075 | 2.396524 | 2.966720 | 2.627568 | 0.347006 | ARDL(1, 3, 1, 0, 2, 1, 0, 1, 2, 0, 1) |
| 193 | -194.078 | 2.396558 | 2.914917 | 2.606597 | 0.338577 | ARDL(1, 0, 2, 1, 0, 1, 0, 1, 2, 0, 1) |
| 194 | -193.08 | 2.396581 | 2.932219 | 2.613622 | 0.341403 | ARDL(1, 0, 1, 2, 0, 2, 0, 1, 2, 0, 1) |
| 195 | -191.088 | 2.396658 | 2.966854 | 2.627702 | 0.346919 | ARDL(1, 2, 1, 0, 2, 1, 0, 1, 2, 1, 1) |
| 196 | -191.088 | 2.396665 | 2.966860 | 2.627708 | 0.346915 | ARDL(1, 2, 1, 0, 2, 1, 1, 1, 2, 0, 1) |
| 197 | -193.089 | 2.396673 | 2.932311 | 2.613714 | 0.341343 | ARDL(1, 0, 1, 0, 0, 2, 0, 2, 2, 1, 1) |
| 198 | -194.09 | 2.396687 | 2.915047 | 2.606726 | 0.338492 | ARDL(1, 0, 1, 0, 1, 1, 0, 2, 2, 0, 1) |
| 199 | -191.093 | 2.396720 | 2.966915 | 2.627763 | 0.346879 | ARDL(1, 2, 1, 0, 2, 2, 0, 1, 2, 0, 1) |
| 200 | -195.094 | 2.396728 | 2.897809 | 2.599766 | 0.335583 | ARDL(1, 0, 1, 1, 0, 0, 0, 2, 2, 0, 1) |

**Table S2.** Possible ARDL candidates

| Model | LogL | AIC* | BIC | HQ | Adj. R^2^ | Specification |
| --- | --- | --- | --- | --- | --- | --- |
| 1 | -201.21 | 2.353299 | 2.697602 | 2.492798 | 0.333393 | ARDL(1, 0, 0, 0, 2, 1) |
| 2 | -202.624 | 2.357703 | 2.684790 | 2.490226 | 0.327294 | ARDL(1, 0, 0, 0, 2, 0) |
| 3 | -200.856 | 2.360172 | 2.721690 | 2.506646 | 0.331921 | ARDL(1, 0, 0, 0, 3, 1) |
| 4 | -200.879 | 2.360410 | 2.721928 | 2.506883 | 0.331763 | ARDL(1, 0, 1, 0, 2, 1) |
| 5 | -201.022 | 2.361934 | 2.723452 | 2.508407 | 0.330743 | ARDL(1, 0, 0, 0, 2, 2) |
| 6 | -201.057 | 2.362313 | 2.723830 | 2.508786 | 0.330490 | ARDL(1, 0, 0, 1, 2, 1) |
| 7 | -201.178 | 2.363596 | 2.725113 | 2.510069 | 0.329630 | ARDL(1, 1, 0, 0, 2, 1) |
| 8 | -202.217 | 2.364012 | 2.708314 | 2.503510 | 0.326213 | ARDL(1, 0, 0, 0, 3, 0) |
| 9 | -202.241 | 2.364269 | 2.708571 | 2.503767 | 0.326040 | ARDL(1, 0, 0, 1, 2, 0) |
| 10 | -200.465 | 2.366653 | 2.745386 | 2.520101 | 0.330685 | ARDL(1, 0, 1, 0, 3, 1) |
| 11 | -202.501 | 2.367037 | 2.711339 | 2.506535 | 0.324172 | ARDL(1, 1, 0, 0, 2, 0) |
| 12 | -200.508 | 2.367106 | 2.745839 | 2.520555 | 0.330382 | ARDL(1, 0, 2, 0, 2, 1) |
| 13 | -202.621 | 2.368312 | 2.712615 | 2.507811 | 0.323309 | ARDL(1, 0, 1, 0, 2, 0) |
| 14 | -200.622 | 2.368324 | 2.747057 | 2.521773 | 0.329566 | ARDL(1, 0, 0, 1, 3, 1) |
| 15 | -203.636 | 2.368471 | 2.695558 | 2.500994 | 0.320012 | ARDL(1, 0, 0, 0, 1, 1) |
| 16 | -200.639 | 2.368500 | 2.747232 | 2.521948 | 0.329448 | ARDL(1, 0, 0, 0, 3, 2) |
| 17 | -200.648 | 2.368591 | 2.747323 | 2.522039 | 0.329387 | ARDL(1, 0, 0, 2, 2, 1) |
| 18 | -200.732 | 2.369484 | 2.748217 | 2.522932 | 0.328788 | ARDL(1, 0, 1, 0, 2, 2) |
| 19 | -201.779 | 2.369994 | 2.731512 | 2.516468 | 0.325327 | ARDL(1, 0, 0, 2, 2, 0) |
| 20 | -200.812 | 2.370337 | 2.749070 | 2.523785 | 0.328215 | ARDL(1, 0, 1, 1, 2, 1) |
| 21 | -200.821 | 2.370434 | 2.749166 | 2.523882 | 0.328150 | ARDL(1, 1, 0, 0, 3, 1) |
| 22 | -200.841 | 2.370654 | 2.749387 | 2.524102 | 0.328002 | ARDL(1, 1, 1, 0, 2, 1) |
| 23 | -200.918 | 2.371471 | 2.750204 | 2.524920 | 0.327453 | ARDL(1, 0, 0, 1, 2, 2) |
| 24 | -201.922 | 2.371515 | 2.733033 | 2.517989 | 0.324300 | ARDL(1, 0, 0, 1, 3, 0) |
| 25 | -204.957 | 2.371888 | 2.681760 | 2.497436 | 0.314443 | ARDL(1, 0, 0, 0, 1, 0) |
| 26 | -201.971 | 2.372028 | 2.733546 | 2.518501 | 0.323954 | ARDL(1, 0, 2, 0, 2, 0) |
| 27 | -200.975 | 2.372072 | 2.750805 | 2.525520 | 0.327049 | ARDL(1, 1, 0, 0, 2, 2) |
| 28 | -200.982 | 2.372151 | 2.750884 | 2.525600 | 0.326995 | ARDL(1, 0, 0, 0, 2, 3) |
| 29 | -200.991 | 2.372243 | 2.750976 | 2.525691 | 0.326933 | ARDL(1, 1, 0, 1, 2, 1) |
| 30 | -200.021 | 2.372562 | 2.768510 | 2.532985 | 0.329806 | ARDL(1, 0, 2, 0, 3, 1) |
| 31 | -202.09 | 2.373299 | 2.734817 | 2.519772 | 0.323094 | ARDL(1, 1, 0, 0, 3, 0) |
| 32 | -201.142 | 2.373852 | 2.752585 | 2.527300 | 0.325850 | ARDL(1, 2, 0, 0, 2, 1) |
| 33 | -202.157 | 2.374008 | 2.735526 | 2.520482 | 0.322614 | ARDL(1, 0, 1, 1, 2, 0) |
| 34 | -202.197 | 2.374432 | 2.735949 | 2.520905 | 0.322327 | ARDL(1, 1, 0, 1, 2, 0) |
| 35 | -202.217 | 2.374650 | 2.736168 | 2.521124 | 0.322179 | ARDL(1, 0, 1, 0, 3, 0) |
| 36 | -200.239 | 2.374885 | 2.770833 | 2.535308 | 0.328247 | ARDL(1, 0, 0, 2, 3, 1) |
| 37 | -203.267 | 2.375178 | 2.719481 | 2.514677 | 0.318647 | ARDL(1, 0, 1, 0, 1, 1) |
| 38 | -200.287 | 2.375392 | 2.771340 | 2.535815 | 0.327907 | ARDL(1, 0, 3, 0, 2, 1) |
| 39 | -200.294 | 2.375472 | 2.771420 | 2.535895 | 0.327853 | ARDL(1, 0, 1, 0, 3, 2) |
| 40 | -200.345 | 2.376015 | 2.771963 | 2.536438 | 0.327488 | ARDL(1, 0, 1, 1, 3, 1) |
| 41 | -203.371 | 2.376284 | 2.720586 | 2.515782 | 0.317894 | ARDL(1, 1, 0, 0, 1, 1) |
| 42 | -204.371 | 2.376288 | 2.703375 | 2.508811 | 0.314675 | ARDL(1, 0, 0, 1, 1, 0) |
| 43 | -200.424 | 2.376851 | 2.772799 | 2.537274 | 0.326925 | ARDL(1, 1, 1, 0, 3, 1) |
| 44 | -200.427 | 2.376887 | 2.772835 | 2.537310 | 0.326901 | ARDL(1, 0, 1, 2, 2, 1) |
| 45 | -200.452 | 2.377149 | 2.773096 | 2.537572 | 0.326725 | ARDL(1, 1, 2, 0, 2, 1) |
| 46 | -200.46 | 2.377237 | 2.773185 | 2.537660 | 0.326666 | ARDL(1, 0, 2, 1, 2, 1) |
| 47 | -200.469 | 2.377327 | 2.773275 | 2.537751 | 0.326605 | ARDL(1, 0, 0, 1, 3, 2) |
| 48 | -201.47 | 2.377335 | 2.756068 | 2.530783 | 0.323497 | ARDL(1, 0, 2, 0, 3, 0) |
| 49 | -202.477 | 2.377413 | 2.738930 | 2.523886 | 0.320303 | ARDL(1, 2, 0, 0, 2, 0) |
| 50 | -201.482 | 2.377473 | 2.756206 | 2.530921 | 0.323404 | ARDL(1, 0, 0, 2, 3, 0) |
| 51 | -200.499 | 2.377646 | 2.773594 | 2.538069 | 0.326390 | ARDL(1, 0, 2, 0, 2, 2) |
| 52 | -202.501 | 2.377670 | 2.739187 | 2.524143 | 0.320129 | ARDL(1, 1, 1, 0, 2, 0) |
| 53 | -204.512 | 2.377787 | 2.704874 | 2.510310 | 0.313647 | ARDL(1, 1, 0, 0, 1, 0) |
| 54 | -200.53 | 2.377978 | 2.773925 | 2.538401 | 0.326167 | ARDL(1, 0, 0, 0, 3, 3) |
| 55 | -200.54 | 2.378084 | 2.774031 | 2.538507 | 0.326095 | ARDL(1, 1, 0, 1, 3, 1) |
| 56 | -201.562 | 2.378321 | 2.757054 | 2.531770 | 0.322830 | ARDL(1, 0, 2, 1, 2, 0) |
| 57 | -200.586 | 2.378579 | 2.774527 | 2.539002 | 0.325761 | ARDL(1, 1, 0, 0, 3, 2) |
| 58 | -203.623 | 2.378966 | 2.723268 | 2.518464 | 0.316062 | ARDL(1, 0, 0, 1, 1, 1) |
| 59 | -200.631 | 2.379054 | 2.775001 | 2.539477 | 0.325441 | ARDL(1, 1, 0, 2, 2, 1) |
| 60 | -200.635 | 2.379093 | 2.775041 | 2.539516 | 0.325415 | ARDL(1, 0, 0, 2, 2, 2) |
| 61 | -203.636 | 2.379108 | 2.723411 | 2.518607 | 0.315964 | ARDL(1, 0, 0, 0, 1, 2) |
| 62 | -200.636 | 2.379112 | 2.775059 | 2.539535 | 0.325402 | ARDL(1, 0, 0, 3, 2, 1) |
| 63 | -200.681 | 2.379583 | 2.775531 | 2.540006 | 0.325084 | ARDL(1, 1, 1, 0, 2, 2) |
| 64 | -201.681 | 2.379589 | 2.758322 | 2.533037 | 0.321971 | ARDL(1, 0, 3, 0, 2, 0) |
| 65 | -200.685 | 2.379626 | 2.775574 | 2.540049 | 0.325055 | ARDL(1, 0, 1, 0, 2, 3) |
| 66 | -200.691 | 2.379687 | 2.775635 | 2.540111 | 0.325013 | ARDL(1, 0, 1, 1, 2, 2) |
| 67 | -201.702 | 2.379808 | 2.758541 | 2.533257 | 0.321822 | ARDL(1, 0, 0, 3, 2, 0) |
| 68 | -201.708 | 2.379873 | 2.758605 | 2.533321 | 0.321778 | ARDL(1, 0, 1, 2, 2, 0) |
| 69 | -199.726 | 2.380060 | 2.793223 | 2.547458 | 0.327834 | ARDL(1, 0, 3, 0, 3, 1) |
| 70 | -200.751 | 2.380332 | 2.776279 | 2.540755 | 0.324578 | ARDL(1, 1, 1, 1, 2, 1) |
| 71 | -201.774 | 2.380575 | 2.759307 | 2.534023 | 0.321302 | ARDL(1, 1, 0, 2, 2, 0) |
| 72 | -206.787 | 2.380715 | 2.673372 | 2.499288 | 0.305056 | ARDL(1, 0, 0, 0, 0, 0) |
| 73 | -200.794 | 2.380792 | 2.776740 | 2.541215 | 0.324268 | ARDL(1, 2, 0, 0, 3, 1) |
| 74 | -201.819 | 2.381053 | 2.759786 | 2.534502 | 0.320977 | ARDL(1, 1, 2, 0, 2, 0) |
| 75 | -200.821 | 2.381078 | 2.777026 | 2.541501 | 0.324074 | ARDL(1, 2, 1, 0, 2, 1) |
| 76 | -201.828 | 2.381148 | 2.759881 | 2.534596 | 0.320913 | ARDL(1, 0, 1, 1, 3, 0) |
| 77 | -200.841 | 2.381283 | 2.777231 | 2.541706 | 0.323936 | ARDL(1, 1, 0, 1, 2, 2) |
| 78 | -201.867 | 2.381569 | 2.760301 | 2.535017 | 0.320627 | ARDL(1, 1, 0, 1, 3, 0) |
| 79 | -200.895 | 2.381862 | 2.777809 | 2.542285 | 0.323544 | ARDL(1, 0, 0, 1, 2, 3) |
| 80 | -200.909 | 2.382010 | 2.777958 | 2.542433 | 0.323444 | ARDL(1, 1, 0, 0, 2, 3) |
| 81 | -199.925 | 2.382181 | 2.795344 | 2.549579 | 0.326406 | ARDL(1, 0, 2, 1, 3, 1) |
| 82 | -199.957 | 2.382519 | 2.795682 | 2.549917 | 0.326179 | ARDL(1, 1, 2, 0, 3, 1) |
| 83 | -204.957 | 2.382526 | 2.709613 | 2.515049 | 0.310387 | ARDL(1, 0, 1, 0, 1, 0) |
| 84 | -200.965 | 2.382606 | 2.778553 | 2.543029 | 0.323041 | ARDL(1, 2, 0, 0, 2, 2) |
| 85 | -200.986 | 2.382829 | 2.778777 | 2.543252 | 0.322890 | ARDL(1, 2, 0, 1, 2, 1) |
| 86 | -199.989 | 2.382865 | 2.796028 | 2.550263 | 0.325945 | ARDL(1, 0, 1, 2, 3, 1) |
| 87 | -202.99 | 2.382870 | 2.744388 | 2.529344 | 0.316584 | ARDL(1, 1, 1, 0, 1, 1) |
| 88 | -200.011 | 2.383096 | 2.796259 | 2.550494 | 0.325790 | ARDL(1, 0, 2, 0, 3, 2) |
| 89 | -202.058 | 2.383601 | 2.762333 | 2.537049 | 0.319245 | ARDL(1, 2, 0, 0, 3, 0) |
| 90 | -203.066 | 2.383684 | 2.745201 | 2.530157 | 0.316028 | ARDL(1, 0, 2, 0, 1, 1) |
| 91 | -201.079 | 2.383818 | 2.779766 | 2.544241 | 0.322219 | ARDL(1, 3, 0, 0, 2, 1) |
| 92 | -202.089 | 2.383924 | 2.762656 | 2.537372 | 0.319025 | ARDL(1, 1, 1, 0, 3, 0) |
| 93 | -201.095 | 2.383992 | 2.779940 | 2.544415 | 0.322102 | ARDL(1, 0, 3, 0, 3, 0) |
| 94 | -202.116 | 2.384211 | 2.762943 | 2.537659 | 0.318830 | ARDL(1, 1, 1, 1, 2, 0) |
| 95 | -204.128 | 2.384342 | 2.728644 | 2.523840 | 0.312375 | ARDL(1, 1, 0, 1, 1, 0) |
| 96 | -200.134 | 2.384408 | 2.797571 | 2.551807 | 0.324904 | ARDL(1, 1, 3, 0, 2, 1) |
| 97 | -201.144 | 2.384507 | 2.780454 | 2.544930 | 0.321753 | ARDL(1, 0, 2, 1, 3, 0) |
| 98 | -200.162 | 2.384707 | 2.797870 | 2.552105 | 0.324702 | ARDL(1, 0, 0, 3, 3, 1) |
| 99 | -200.166 | 2.384743 | 2.797906 | 2.552141 | 0.324679 | ARDL(1, 0, 1, 0, 3, 3) |
| 100 | -206.177 | 2.384856 | 2.694729 | 2.510405 | 0.305494 | ARDL(1, 0, 0, 0, 0, 1) |
| 101 | -202.192 | 2.385019 | 2.763752 | 2.538467 | 0.318279 | ARDL(1, 2, 0, 1, 2, 0) |
| 102 | -204.194 | 2.385041 | 2.729344 | 2.524540 | 0.311894 | ARDL(1, 0, 1, 1, 1, 0) |
| 103 | -200.211 | 2.385227 | 2.798390 | 2.552625 | 0.324351 | ARDL(1, 0, 1, 1, 3, 2) |
| 104 | -200.213 | 2.385244 | 2.798407 | 2.552642 | 0.324340 | ARDL(1, 1, 0, 2, 3, 1) |
| 105 | -200.235 | 2.385476 | 2.798639 | 2.552874 | 0.324183 | ARDL(1, 0, 0, 2, 3, 2) |
| 106 | -200.237 | 2.385502 | 2.798665 | 2.552900 | 0.324165 | ARDL(1, 1, 1, 0, 3, 2) |
| 107 | -200.246 | 2.385591 | 2.798754 | 2.552989 | 0.324106 | ARDL(1, 0, 3, 1, 2, 1) |
| 108 | -203.265 | 2.385798 | 2.747316 | 2.532271 | 0.314580 | ARDL(1, 0, 1, 0, 1, 2) |
| 109 | -203.266 | 2.385808 | 2.747325 | 2.532281 | 0.314573 | ARDL(1, 0, 1, 1, 1, 1) |
| 110 | -200.269 | 2.385844 | 2.799007 | 2.553242 | 0.323934 | ARDL(1, 1, 1, 1, 3, 1) |
| 111 | -204.281 | 2.385971 | 2.730273 | 2.525469 | 0.311254 | ARDL(1, 0, 0, 2, 1, 0) |
| 112 | -201.282 | 2.385978 | 2.781925 | 2.546401 | 0.320754 | ARDL(1, 0, 3, 1, 2, 0) |
| 113 | -200.287 | 2.386030 | 2.799193 | 2.553428 | 0.323809 | ARDL(1, 0, 3, 0, 2, 2) |
| 114 | -201.29 | 2.386069 | 2.782017 | 2.546492 | 0.320692 | ARDL(1, 0, 0, 3, 3, 0) |
| 115 | -201.305 | 2.386227 | 2.782175 | 2.546650 | 0.320585 | ARDL(1, 1, 2, 0, 3, 0) |
| 116 | -200.306 | 2.386236 | 2.799399 | 2.553634 | 0.323669 | ARDL(1, 0, 2, 2, 2, 1) |
| 117 | -203.315 | 2.386335 | 2.747852 | 2.532808 | 0.314212 | ARDL(1, 1, 0, 1, 1, 1) |
| 118 | -202.34 | 2.386596 | 2.765329 | 2.540044 | 0.317203 | ARDL(1, 3, 0, 0, 2, 0) |
| 119 | -206.344 | 2.386633 | 2.696505 | 2.512182 | 0.304259 | ARDL(1, 1, 0, 0, 0, 0) |
| 120 | -201.364 | 2.386854 | 2.782801 | 2.547277 | 0.320159 | ARDL(1, 1, 3, 0, 2, 0) |
| 121 | -203.364 | 2.386854 | 2.748372 | 2.533328 | 0.313856 | ARDL(1, 1, 0, 0, 1, 2) |
| 122 | -203.371 | 2.386922 | 2.748440 | 2.533395 | 0.313809 | ARDL(1, 2, 0, 0, 1, 1) |
| 123 | -200.373 | 2.386944 | 2.800107 | 2.554342 | 0.323190 | ARDL(1, 1, 0, 1, 3, 2) |
| 124 | -200.381 | 2.387028 | 2.800191 | 2.554426 | 0.323134 | ARDL(1, 1, 2, 1, 2, 1) |
| 125 | -200.39 | 2.387126 | 2.800289 | 2.554524 | 0.323067 | ARDL(1, 0, 0, 1, 3, 3) |
| 126 | -201.402 | 2.387253 | 2.783201 | 2.547676 | 0.319887 | ARDL(1, 0, 1, 2, 3, 0) |
| 127 | -200.411 | 2.387353 | 2.800516 | 2.554751 | 0.322914 | ARDL(1, 0, 1, 2, 2, 2) |
| 128 | -200.412 | 2.387365 | 2.800528 | 2.554763 | 0.322905 | ARDL(1, 2, 1, 0, 3, 1) |
| 129 | -200.413 | 2.387370 | 2.800533 | 2.554768 | 0.322902 | ARDL(1, 1, 1, 2, 2, 1) |
| 130 | -204.416 | 2.387400 | 2.731702 | 2.526898 | 0.310269 | ARDL(1, 2, 0, 0, 1, 0) |
| 131 | -200.422 | 2.387471 | 2.800633 | 2.554869 | 0.322834 | ARDL(1, 0, 1, 3, 2, 1) |
| 132 | -200.428 | 2.387537 | 2.800700 | 2.554935 | 0.322789 | ARDL(1, 1, 0, 0, 3, 3) |
| 133 | -201.435 | 2.387606 | 2.783554 | 2.548029 | 0.319647 | ARDL(1, 0, 2, 2, 2, 0) |
| 134 | -200.437 | 2.387623 | 2.800786 | 2.555021 | 0.322730 | ARDL(1, 0, 2, 0, 2, 3) |
| 135 | -200.44 | 2.387659 | 2.800822 | 2.555057 | 0.322706 | ARDL(1, 1, 2, 0, 2, 2) |
| 136 | -200.45 | 2.387767 | 2.800930 | 2.555165 | 0.322633 | ARDL(1, 2, 2, 0, 2, 1) |
| 137 | -200.456 | 2.387828 | 2.800991 | 2.555226 | 0.322592 | ARDL(1, 0, 2, 1, 2, 2) |
| 138 | -201.472 | 2.388005 | 2.783953 | 2.548428 | 0.319376 | ARDL(1, 1, 0, 2, 3, 0) |
| 139 | -202.477 | 2.388050 | 2.766782 | 2.541498 | 0.316210 | ARDL(1, 2, 1, 0, 2, 0) |
| 140 | -201.498 | 2.388277 | 2.784224 | 2.548700 | 0.319191 | ARDL(1, 1, 2, 1, 2, 0) |
| 141 | -204.51 | 2.388401 | 2.732703 | 2.527899 | 0.309579 | ARDL(1, 1, 1, 0, 1, 0) |
| 142 | -199.538 | 2.388707 | 2.819085 | 2.563080 | 0.325055 | ARDL(1, 1, 3, 0, 3, 1) |
| 143 | -200.54 | 2.388721 | 2.801884 | 2.556119 | 0.321986 | ARDL(1, 2, 0, 1, 3, 1) |
| 144 | -204.541 | 2.388735 | 2.733037 | 2.528234 | 0.309348 | ARDL(1, 0, 2, 0, 1, 0) |
| 145 | -200.556 | 2.388893 | 2.802056 | 2.556291 | 0.321870 | ARDL(1, 0, 0, 2, 2, 3) |
| 146 | -203.566 | 2.388996 | 2.750514 | 2.535470 | 0.312384 | ARDL(1, 0, 0, 2, 1, 1) |
| 147 | -200.582 | 2.389175 | 2.802338 | 2.556573 | 0.321679 | ARDL(1, 2, 0, 0, 3, 2) |
| 148 | -200.605 | 2.389410 | 2.802573 | 2.556808 | 0.321520 | ARDL(1, 1, 1, 0, 2, 3) |
| 149 | -200.617 | 2.389546 | 2.802709 | 2.556944 | 0.321427 | ARDL(1, 2, 0, 2, 2, 1) |
| 150 | -200.62 | 2.389574 | 2.802737 | 2.556972 | 0.321408 | ARDL(1, 1, 1, 1, 2, 2) |
| 151 | -203.622 | 2.389594 | 2.751111 | 2.536067 | 0.311974 | ARDL(1, 0, 0, 0, 1, 3) |
| 152 | -203.623 | 2.389602 | 2.751119 | 2.536075 | 0.311968 | ARDL(1, 0, 0, 1, 1, 2) |
| 153 | -200.623 | 2.389604 | 2.802767 | 2.557002 | 0.321387 | ARDL(1, 0, 0, 3, 2, 2) |
| 154 | -200.623 | 2.389604 | 2.802767 | 2.557002 | 0.321387 | ARDL(1, 1, 0, 3, 2, 1) |
| 155 | -200.625 | 2.389625 | 2.802788 | 2.557023 | 0.321373 | ARDL(1, 1, 0, 2, 2, 2) |
| 156 | -201.636 | 2.389746 | 2.785694 | 2.550169 | 0.318190 | ARDL(1, 0, 1, 3, 2, 0) |
| 157 | -199.636 | 2.389749 | 2.820127 | 2.564122 | 0.324352 | ARDL(1, 0, 3, 1, 3, 1) |
| 158 | -200.656 | 2.389958 | 2.803121 | 2.557356 | 0.321147 | ARDL(1, 0, 1, 1, 2, 3) |
| 159 | -206.663 | 2.390031 | 2.699904 | 2.515580 | 0.301891 | ARDL(1, 0, 0, 1, 0, 0) |
| 160 | -200.677 | 2.390178 | 2.803341 | 2.557576 | 0.320998 | ARDL(1, 2, 1, 0, 2, 2) |
| 161 | -201.7 | 2.390426 | 2.786373 | 2.550849 | 0.317726 | ARDL(1, 1, 0, 3, 2, 0) |
| 162 | -201.704 | 2.390463 | 2.786411 | 2.550886 | 0.317701 | ARDL(1, 1, 1, 2, 2, 0) |
| 163 | -200.706 | 2.390492 | 2.803655 | 2.557890 | 0.320785 | ARDL(1, 3, 1, 0, 2, 1) |
| 164 | -199.726 | 2.390698 | 2.821076 | 2.565071 | 0.323710 | ARDL(1, 0, 3, 0, 3, 2) |
| 165 | -200.73 | 2.390746 | 2.803909 | 2.558144 | 0.320612 | ARDL(1, 1, 3, 0, 3, 0) |
| 166 | -202.742 | 2.390868 | 2.769600 | 2.544316 | 0.314280 | ARDL(1, 1, 2, 0, 1, 1) |
| 167 | -200.747 | 2.390928 | 2.804091 | 2.558326 | 0.320489 | ARDL(1, 2, 1, 1, 2, 1) |
| 168 | -201.755 | 2.391012 | 2.786960 | 2.551436 | 0.317326 | ARDL(1, 2, 2, 0, 2, 0) |
| 169 | -201.756 | 2.391022 | 2.786970 | 2.551445 | 0.317319 | ARDL(1, 2, 0, 2, 2, 0) |
| 170 | -200.764 | 2.391107 | 2.804270 | 2.558505 | 0.320367 | ARDL(1, 3, 0, 0, 3, 1) |
| 171 | -206.773 | 2.391202 | 2.701074 | 2.516750 | 0.301074 | ARDL(1, 0, 1, 0, 0, 0) |
| 172 | -201.777 | 2.391247 | 2.787194 | 2.551670 | 0.317166 | ARDL(1, 1, 1, 1, 3, 0) |
| 173 | -200.785 | 2.391334 | 2.804497 | 2.558732 | 0.320213 | ARDL(1, 0, 3, 1, 3, 0) |
| 174 | -200.794 | 2.391422 | 2.804585 | 2.558820 | 0.320153 | ARDL(1, 1, 0, 1, 2, 3) |
| 175 | -199.812 | 2.391622 | 2.821999 | 2.565994 | 0.323085 | ARDL(1, 0, 2, 2, 3, 1) |
| 176 | -203.821 | 2.391710 | 2.753227 | 2.538183 | 0.310516 | ARDL(1, 0, 2, 1, 1, 0) |
| 177 | -199.824 | 2.391744 | 2.822122 | 2.566117 | 0.323002 | ARDL(1, 1, 2, 1, 3, 1) |
| 178 | -200.841 | 2.391921 | 2.805084 | 2.559319 | 0.319813 | ARDL(1, 2, 0, 1, 2, 2) |
| 179 | -199.847 | 2.391988 | 2.822366 | 2.566361 | 0.322837 | ARDL(1, 0, 2, 0, 3, 3) |
| 180 | -205.855 | 2.392070 | 2.719157 | 2.524593 | 0.303774 | ARDL(1, 1, 0, 0, 0, 1) |
| 181 | -205.862 | 2.392149 | 2.719236 | 2.524672 | 0.303719 | ARDL(1, 0, 1, 0, 0, 1) |
| 182 | -201.867 | 2.392204 | 2.788152 | 2.552627 | 0.316512 | ARDL(1, 2, 0, 1, 3, 0) |
| 183 | -200.895 | 2.392500 | 2.805663 | 2.559898 | 0.319420 | ARDL(1, 2, 0, 0, 2, 3) |
| 184 | -200.904 | 2.392592 | 2.805755 | 2.559990 | 0.319357 | ARDL(1, 3, 0, 1, 2, 1) |
| 185 | -199.922 | 2.392785 | 2.823163 | 2.567158 | 0.322297 | ARDL(1, 0, 2, 1, 3, 2) |
| 186 | -199.928 | 2.392856 | 2.823233 | 2.567228 | 0.322249 | ARDL(1, 0, 1, 3, 3, 1) |
| 187 | -200.935 | 2.392930 | 2.806093 | 2.560328 | 0.319127 | ARDL(1, 3, 0, 0, 2, 2) |
| 188 | -199.944 | 2.393021 | 2.823398 | 2.567393 | 0.322138 | ARDL(1, 1, 2, 0, 3, 2) |
| 189 | -202.947 | 2.393055 | 2.771788 | 2.546504 | 0.312778 | ARDL(1, 0, 3, 0, 1, 1) |
| 190 | -199.957 | 2.393157 | 2.823535 | 2.567530 | 0.322045 | ARDL(1, 2, 2, 0, 3, 1) |
| 191 | -199.965 | 2.393247 | 2.823625 | 2.567620 | 0.321984 | ARDL(1, 1, 1, 2, 3, 1) |
| 192 | -203.97 | 2.393299 | 2.754817 | 2.539773 | 0.309419 | ARDL(1, 1, 1, 1, 1, 0) |
| 193 | -202.973 | 2.393327 | 2.772059 | 2.546775 | 0.312592 | ARDL(1, 0, 2, 0, 1, 2) |
| 194 | -201.978 | 2.393384 | 2.789332 | 2.553807 | 0.315705 | ARDL(1, 3, 0, 0, 3, 0) |
| 195 | -202.982 | 2.393421 | 2.772153 | 2.546869 | 0.312527 | ARDL(1, 1, 1, 1, 1, 1) |
| 196 | -199.983 | 2.393435 | 2.823813 | 2.567808 | 0.321856 | ARDL(1, 0, 1, 2, 3, 2) |
| 197 | -202.987 | 2.393477 | 2.772210 | 2.546925 | 0.312489 | ARDL(1, 2, 1, 0, 1, 1) |
| 198 | -202.989 | 2.393499 | 2.772231 | 2.546947 | 0.312474 | ARDL(1, 1, 1, 0, 1, 2) |
| 199 | -204.026 | 2.393890 | 2.755408 | 2.540364 | 0.309011 | ARDL(1, 1, 2, 0, 1, 0) |
| 200 | -206.048 | 2.394133 | 2.721220 | 2.526656 | 0.302336 | ARDL(1, 0, 0, 1, 0, 1) |
